# Geographic structure in the Southern Ocean circumpolar brittle star *Ophionotus victoriae* (Ophiuridae) revealed from mtDNA and single‐nucleotide polymorphism data

**DOI:** 10.1002/ece3.2617

**Published:** 2016-12-16

**Authors:** Matthew P. Galaska, Chester J. Sands, Scott R. Santos, Andrew R. Mahon, Kenneth M. Halanych

**Affiliations:** ^1^ Department of Biological SciencesAuburn UniversityAuburnALUSA; ^2^ Natural Environment Research CouncilBritish Antarctic SurveyCambridgeUK; ^3^ Department of BiologyCentral Michigan UniversityMount PleasantMIUSA

**Keywords:** Antarctica, cytochrome c oxidase subunit I, ophiuroid, phylogeography, population genetics, restriction‐associated DNA, single‐nucleotide polymorphism

## Abstract

Marine systems have traditionally been thought of as “open” with few barriers to gene flow. In particular, many marine organisms in the Southern Ocean purportedly possess circumpolar distributions that have rarely been well verified. Here, we use the highly abundant and endemic Southern Ocean brittle star *Ophionotus victoriae* to examine genetic structure and determine whether barriers to gene flow have existed around the Antarctic continent. *Ophionotus victoriae* possesses feeding planktotrophic larvae with presumed high dispersal capability, but a previous study revealed genetic structure along the Antarctic Peninsula. To test the extent of genetic differentiation within *O. victoriae*, we sampled from the Ross Sea through the eastern Weddell Sea. Whereas two mitochondrial DNA markers (16S rDNA and COI) were employed to allow comparison to earlier work, a 2b‐RAD single‐nucleotide polymorphism (SNP) approach allowed sampling of loci across the genome. Mitochondrial data from 414 individuals suggested three major lineages, but 2b‐RAD data generated 1,999 biallelic loci that identified four geographically distinct groups from 89 samples. Given the greater resolution by SNP data, *O. victoriae* can be divided into geographically distinct populations likely representing multiple species. Specific historical scenarios that explain current population structure were examined with approximate Bayesian computation (ABC) analyses. Although the Bransfield Strait region shows high diversity possibly due to mixing, our results suggest that within the recent past, dispersal processes due to strong currents such as the Antarctic Circumpolar Current have not overcome genetic subdivision presumably due to historical isolation, questioning the idea of large open circumpolar populations in the Southern Ocean.

## 1 Introduction

The Southern Ocean (SO) is characterized by rich biodiversity and largely endemic benthic fauna (Kaiser et al., 2013), resulting from an active geological history and organismal adaption to an extreme environment. While the Antarctic Polar Front (APF) serves to isolate the SO from warmer waters at lower latitudes, the Antarctic Circumpolar Current (ACC) is the world's strongest major current that has been presumed to aid dispersal of many marine species within the SO (Bathmann, Scharek, Klaas, Dubischarr, & Smetacek, 1997; Smetacek, De Baar, Bathmann, Lochte, & Rutgers Van Der Loeff, 1997; Thornhill, Mahon, Norenburg, & Halanych, 2008). The fact that the ACC can promote long‐distance dispersal has helped reinforce the historically held assumption that many marine organisms of the SO likely have a circumpolar distribution around Antarctica (Dayton, Mordida, & Bacon, 1994). Antarctic currents closer to shore such as the Circumpolar Deep Water, Ross Gyre, and Weddell Gyre add complexity in predicting geographic dispersal capabilities of species (Tynan, 1998).

In addition to dispersal mediated by oceanic currents, glaciation cycles have also played a role in Antarctic biodiversity through controlling habitat availability (Thatje, Hillenbrand, & Larter, 2005). Glacial maximums during the Cenozoic likely forced species into the deep sea with pockets of refugia on the shelf allowing some species to recolonize and ultimately shape the SO's current community structure (Thatje et al., 2005). Polynyas, open regions of water surrounded by sea ice, in the SO may also serve as areas of refuge and often contribute higher levels of primary production (Massom & Stammerjohn, 2010). In expansion phases, grounded ice sheets can physically cover large geographic areas of the continental shelf, displacing inhabitants, and physically reshaping environments by removal and rearrangement of benthic habitat. During glacial contraction, new habitat becomes available allowing for population expansion. Thus, glacial cycles can drive population fragmentation and expansion opportunities (Thatje et al., 2005), ultimately serving as a biological “diversity pump” (Clarke & Crame, 1992).

**Figure 1:**
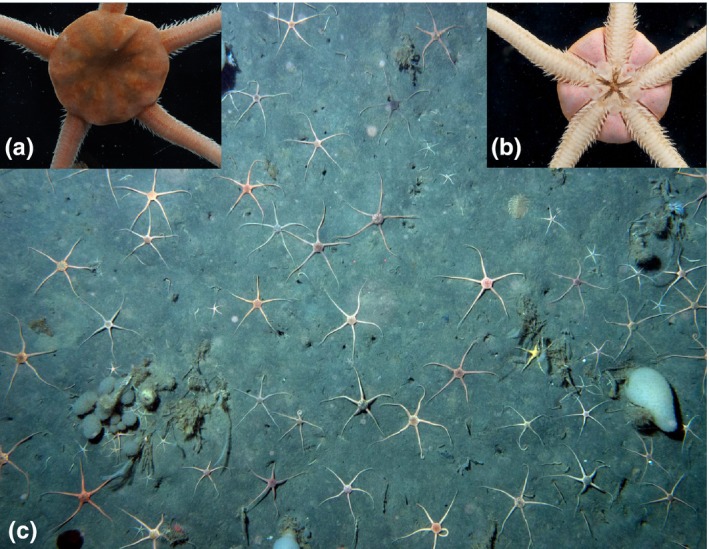
(a) Aboral view of *Ophionotus victoriae*. (b) Oral view of *O. victoriae*. (c) Yo‐Yo camera image of SO benthic ecosystem consisting of many ophiuroid species including the dominant *O. victoriae*. This image was taken at a depth of 313 m, near Anderson Island at the south end of Antarctic Sound (−63°40′42.0″S 56°14′18.0″W). Photographs (a) and (b) kindly provided by Dr. Christoph Held [Correction added on 05 January 2017: Figure 1 has been updated in this version.]

Brittle stars are important members of SO biodiversity, comprising at least 219 nominal species and 126 that are endemic from the region (Martín‐Ledo & López‐González, 2014). Three of these species belong to *Ophionotus* (*O. hexactis* (Smith 1876), *O. taylori* McKnight, 1967; and *O. victoriae* Bell 1902); all of which also occur in the SO but are morphologically distinct from each other. *Ophionotus victoriae* Bell, 1902 is the most common and is a highly abundant (Figure 1), conspicuous ophiuroid endemic to the SO. This species has been reported to have a circumpolar distribution (Fell, 1961) and occupies many different benthic substrates within Antarctic waters (Fratt & Dearborn, 1984), with the South Sandwich Islands as its northern most limit (Sands et al., 2012). *Ophionotus victoriae* has a long‐lived, planktotrophic larvae, remaining in the water column for several months (Pearse, McClintock, & Bosch, 1991), thus allowing for the possibility of long‐distance dispersal via the ACC. Previous phylogeographic work using the mitochondrial DNA (mtDNA) 16S ribosomal subunit (16S) and cytochrome c oxidase subunit I (COI) gene fragments reported unexpected levels of genetic diversity and divergence along the Antarctic Peninsula and oceanic islands (South Sandwich Islands and Bouvet Island), suggesting *O. victoriae* possesses higher‐than‐expected geographic structure and questions the possibility of cryptic species (Hunter & Halanych, 2010). Given this, a larger sampling effort around Antarctica would likely result in the uncovering of additional diversity and potential discovery of cryptic species.

To test for phylogeographic structure in this supposed circumpolar species, and to provide insight on processes of dispersal and historical isolation, molecular tools were used to examine *O. victoriae* over a >7,000 km range from the Western Ross Sea to the eastern Weddell. This study, to the best of our knowledge, also includes the first sampling of benthic invertebrates from Wrights Bay, located between the Amundsen and Ross Seas. Herein, we utilized the mitochondrial 16S and COI genes to allow direct comparisons to results of Hunter and Halanych (2010) as well as a high‐resolution whole‐genome single‐nucleotide polymorphism (SNP)‐based approach, specifically 2b‐RAD (Wang, Meyer, McKay, & Matz, 2012). This latter approach was chosen as restriction‐associated DNA (RAD)‐tags have been shown to identify fine‐scale population structure in marine species beyond the resolution of mtDNA genes (Benestan et al., 2015; Reitzel, Herrera, Layden, Martindale, & Shank, 2013). Assessing population structure for organisms like *O. victoriae* of the SO is also important toward anticipating changes in the Antarctic benthic ecosystem as species ranges and structure will likely shift with future climate change (Aronson et al., 2007).

## 2 Methods

### 2.1 Sample collection

**Figure 2:**
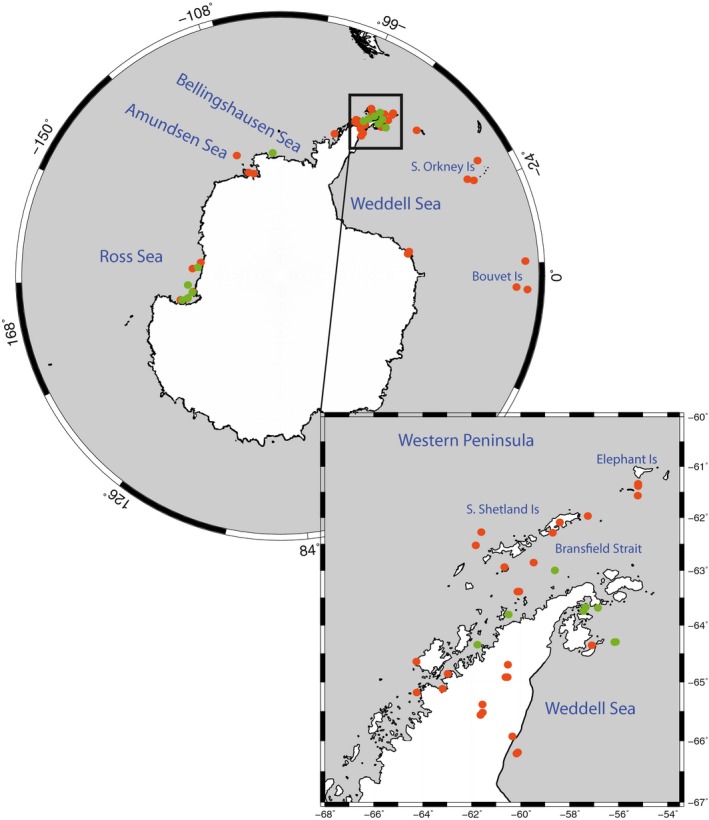
Distribution of *Ophionotus victoriae*. Green dots represent sampling localities with 2b‐RAD and mtDNA data, while orange dots represent localities where solely mtDNA was utilized. Due to the proximity of some localities, overlap on the map could not be avoided. Sampling localities in the Antarctic Peninsula inset that appear to be on land represent locations now open to the sea since the Larsen Ice Shelf broke away

Specimens of *O. victoriae* were collected during four National Science Foundation (NSF)‐sponsored research expeditions (*RVIB Nathaniel B. Palmer* 12‐10, *RV Laurence M. Gould* 04‐14, 06‐05 & 13‐12), three British Antarctic Survey (BAS)‐sponsored expeditions (*RRS James Clark Ross* JR144, JR179, and JR230), and from an Alfred Wegener Institute (AWI) campaign (*RV Polarstern* PS77). Upon collection, samples were morphologically examined (mainly by MPG, CJS, and KMH) to verify species designations as described (McKnight, 1967; Sieg & Waegele, 1990). Oceanic island samples used in Hunter and Halanych (2010) were kindly made available from the NSF IceFish cruise and W. Deitrich (OPP‐0132032). In total, the mitochondrial dataset included 414 specimens over 88 sampling localities that span the Ross, Amundsen, Bellingshausen, Antarctic Peninsula, Weddell Seas and oceanic islands, or a geographic distance of >7,000 km (Figure 2 and Table S1). Samples available for 2b‐RAD analyses included 96 specimens from 15 sampling localities ranging from the Ross Sea to the western portion of the Weddell Sea, a geographic distance >5,000 km.

### 2.2 Data collection

Genomic DNA was extracted using Qiagen's DNeasy^®^ blood and tissue kit following the manufacturer's protocol. Extracted DNA was utilized in amplification of two mtDNA fragments from the COI and 16S genes. Because COI sequences typically provide considerably more resolution then 16S data (Mahon, Arango, & Halanych, 2008; Thornhill et al., 2008; Wilson, Schrödl, & Halanych, 2009), we allocated resources to maximize the number of individuals sampled for COI. A ~560‐bp fragment of COI was amplified for 414 samples with the Folmer COI primer set (Folmer, Black, Hoeh, Lutz, & Vrijenhoek, 1994) LCO1490 (5′‐GGTCAACAAATCATAAAGATATTGG‐3′) and HCO2198 (5′‐TAAACTTCAGGGTGACCAAAAAATCA‐3′). Polymerase chain reaction (PCR) cycling conditions for COI were as follows: initial denaturation at 94°C for 3 min; 40 cycles of denaturation at 94°C for 30 s; annealing at 51°C for 1 min; extension at 72°C for 1 min; and final extension at 72°C for 2 min. Additionally, a ~500‐bp fragment was amplified for 252 samples using the 16S primer set (Palumbi, 2007) 16SarL (5′‐CGCCTGTTTATCAAAAACAT‐3′) and 16SbrH (5′‐CCGGTCTGAACTCAGATCACGT‐3′). PCR cycling conditions employed for 16S were as follows: initial denaturation at 94°C for 3 min; 35 cycles of denaturation at 94°C for 30 s; annealing at 46°C for 30 s; extension at 72°C for 30 s; and final extension at 72°C for 3 min. Amplicons for the COI and 16S genes were sent to Genewiz, Inc. (South Plainfield, NJ, USA) for bidirectional Sanger sequencing. Chromatograms were assembled and edited using Sequencher^®^ 5.4 (Gene Codes, Ann Arbor, MI, USA), and finished sequences were aligned with MEGA 6 (Tamura, Stecher, Peterson, Filipski, & Kumar, 2013). Analyses of molecular variance (AMOVA) were performed with Arlequin 3.5.1.2 (Excoffier, Laval, & Schneider, 2005) to test for genetic differentiation between sampling localities by geographic regions (i.e., the Ross Sea, Bellingshausen Sea–Amundsen Sea, Western Peninsula, Weddell Sea, and oceanic islands). Sampling information for AMOVA including pooling scheme can be located in Figure S1. TCS analyses (Templeton, Crandall, & Sing, 1992) were used to reconstruct statistical parsimony networks as implemented in PopART (Leigh & Bryant, 2015) (http://popart.otago.ac.nz) for COI (414 samples), 16S (251 samples), and a concatenation of both mtDNA fragments (251 samples).

To aid with species delineation based on CO1 sequence data, a histogram of uncorrected pairwise distances (p) (Craft et al., 2008) was generated comparing all unique haplotypes of *O. victoriae* (Table S8), *Ophionotus hexactis* (GenBank Accession Number KU895454.1), and *Ophiacantha spectabilis* (EU869959.1‐EU869961.1). The latter taxa were employed to reveal genetic distance to other related ophiuroid taxa.

For RAD‐tag analyses, a subset of 96 samples spanning 15 sampling locations from the Ross through the western Weddell Seas were examined. Due to logistical issues, only samples from the *RVIB Nathaniel B. Palmer* 12‐10 and *RV Laurence M. Gould* 13‐12 cruises were available for 2b‐RAD processing. Samples were prepared following Wang et al.'s (2012) 2b‐RAD protocol with the restriction enzyme *Alf*I. To avoid potential issues with PCR inhibitors in *O. victoriae*, samples were extracted using Qiagen's DNeasy^®^ Plant Mini Kit. Selection of an appropriate reduction scheme was carried out by utilizing the genome size of *Ophioplocus esmarki* (*C*‐value = 3.00) (Hinegardner, 1974) as a proxy as it is the most closely related ophiuroid to *O. victoriae* for which such information was available. Due to the large estimated genome size of *O. victoriae*, samples were prepped and dual‐barcoded targeting a reduced subset of *Alf*I sites through a 1/32nd reduction scheme to target roughly 2,000 SNPs. Sequencing was performed at the Genome Services Laboratory at HudsonAlpha Institute for Biotechnology (Huntsville, Alabama) on an Illumina Hi‐Seq 2000 using v4 chemistry and generating 50‐bp single‐end reads.

Raw Illumina reads were demultiplexed by sample, quality‐filtered, and aligned against a custom‐derived de novo reference following bioinformatic steps outlined in Wang et al.'s (2012) 2b‐RAD protocol and with scripts provided by Dr. Eli Meyer (Oregon State University) (https://github.com/Eli-Meyer). Specifically, data were first filtered by loci with a minimum coverage of 25X. For all SNPs, loci scored as homozygotic were defined to have a maximum variance of 1%, whereas those that were consider heterozygotic had a minimum of 25% variance. Loci deviating from these definitions were excluded from further analyses. Remaining SNP loci were then further filtered to only include loci that were present in ≥80% of individuals. To ensure individuals with large amounts of missing data did not skew analyses, those with ≤80% of the remaining SNP loci were also removed from the dataset. Raw data were also processed and analyzed using the software *Stacks* (Catchen, Amores, Hohenlohe, Cresko, & Postlethwait, 2011), but both methods yielded similar interpretation of data and evolutionary patterns and processes.

To determine the potential number of populations (*K*), STRUCTURE 2.3.4 (Pritchard, Stephens, & Donnelly, 2000) was utilized with the following parameters: (1) seven replicates at each potential *K* (1–15); (2) an admixture model with correlated allele frequencies; (3) a 50,000 repetition burn‐in period; and (4) 100,000 additional Markov chain Monte Carlo (MCMC) repetitions. Because SNP datasets can vary in λ (parameter around the allele frequency prior) compared to mtDNA (Pritchard, Wen, & Falush, 2010), an initial run was used to infer λ to be 0.2447 prior to the full run. Resulting files were then processed with STRUCTURE HARVESTER (Earl & VonHoldt, 2012) to determine the most likely values of *K* from Delta *K* analyses as well as CLUMPP (Jakobsson & Rosenberg, 2007), and DISTRUCT (Rosenberg, 2004) to visualize the *K* outputs.

Because results for *K* from STRUCTURE varied by MCMC run and seemed inconsistent with biological knowledge of *O. victoriae*, we used additional approaches to estimate *K*. SMARTPCA (Patterson, Price, & Reich, 2006) was used to further validate population structure by performing principal component analyses (PCAs) in the EIGENSOFT software package (Price et al., 2006). Samples were analyzed and labeled by both STRUCTURE *K* results and geographic region for PCAs. Geographic regions for PCAs include Ross Sea, Bellingshausen Sea, western Antarctic Peninsula, Bransfield Strait, and Weddell Sea. One sampling locality in the Bransfield Strait (Op877) was likely an intermixing site based on results of STRUCTURE pairwise *F*_ST_ and PCA. Thus, analyses were performed considering Op877 as belonging to both possible source populations. This did not affect interpretation of results, and thus, samples from Op877 were pooled with the population for which it is the most similar, the Weddell‐A population. Furthermore, BayeScan (Foll & Gaggiotti, 2008) was utilized with four threads, 100 runs at a 100,000 burn‐in length and 100,000 pilot length to identify any loci that might be under selection, and molecular diversity analyses were performed using GENEPOP (Rousset, 2008).

DIYABC v2.0 (Cornuet et al., 2014) was used for an approximate Bayesian computation (ABC) analyses (Beaumont, Zhang, & Balding, 2002) to evaluate historical geographic patterns of divergence. This was achieved in DIYABC v2.0 by calculating summary statistics from prior distribution models in each proposed scenario. Specifically, seven different scenarios based off Antarctic currents and geographic history were evaluated to test whether the population structure was due to glacial refugia, current‐mediated gene flow, or localized restriction of gene flow. All seven historical scenarios tested are shown and described in Figure S1. Input populations needed for DIYABC v2.0 analyses were selected based off results of STRUCTURE analyses. DIYABC v2.0 uses principal component analyses (PCAs) to determine the confidence in each scenario and priors.

All sequences collected herein are reported under GenBank accession numbers KY048203‐KY048268. Raw reads for 2b‐RAD SNP data are deposited to NCBI Sequence Read Archive (SRA) accession numbers SAMN05944630‐SAMN5944718. Data matrices and alignments are deposited to Dryad under accession numbers doi:10.5061/dryad.0k1r0.

## 3 Results

### 3.1 Mitochondrial

**Figure 3:**
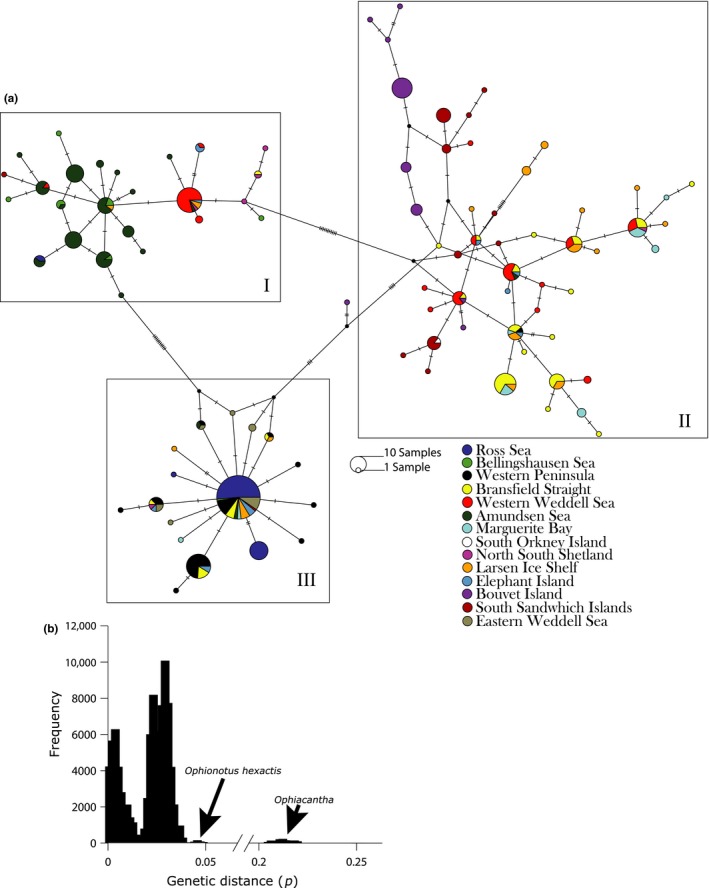
(a) Haplotype network of *Ophionotus victoriae* produced by PopART (Leigh & Bryant, 2015). The haplotype network is based off COI data from a TCS1.21 (Clement, Posada, & Crandall, 2000) analyses of 414 samples. Filled black dots represent missing haplotypes. In addition, maximum‐likelihood analyses also revealed three clades. (b) Histogram of COI uncorrected pairwise distances (*p*)

Both COI and 16S mitochondrial fragments revealed genetic structure within *O. victoriae*. COI data analyzed from 414 individual yielded an increased nucleotide diversity from an extended geographic range in comparison with 16S or concatenated COI and 16S data for 252 individuals (Table 1). Thus, the following discussion focuses mainly on COI results as this marker provided more phylogeographic signal. Analyses of 16S and concatenated dataset are more fully reported in Supplementary Materials. Tests for selection via Tajima's *D* were negative, but not significant (*p* > .10), for both mitochondrial markers (Table 1). AMOVA results for COI data with groupings defined by geographic regions (i.e., Ross Sea, Bellingshausen Sea–Amundsen Sea, Western Peninsula, Weddell Sea, and oceanic islands) revealed 37.87% of the molecular variation as occurring between geographic regions (Table 2). Additionally, three major lineages were recovered in the parsimony network analysis of COI (Figure 3a), primarily corresponding to the following geographic regions: (I) Amundsen and Bellingshausen Seas with some individuals from the western Weddell Sea; (II) the western Weddell Sea with oceanic islands; and (III) the Ross Sea with the eastern Weddell Sea and Western Antarctic Peninsula. To show how the lineages relate to one another, the network was kept whole but applying a 95% connection limit will separate genetic lineage I into its own unconnected network.

**Table 1:** Standard nucleotide indices from mtDNA. Tajima's *D* was found to be not significant in all analyses

	COI	16S	COI &16S
Number of samples	414	251	251
Nucleotide diversity	0.0179446	0.00394178	0.00989389
Segregating sites	67	22	73
Parsimony‐informative sites	45	14	51
Tajima's *D*	−0.38834, *p *> .10	−1.23336, *p* > .10	−0.411972, *p *> .10

**Table 2:** Analysis of molecular variance statistics for *Ophionotus victoriae* based on COI data

Source of variation	*df*	Sum of squares	σ^2^	Percentage of variation
Among groups	4	13832.777	30.561	37.87802
Among populations within groups	6	2666.908	17.744	21.99185
Within populations	403	13048.374	32.378	40.13013
Total	413	29548.06	80.638	

The histogram of uncorrected pairwise distances (Figure 3b) yielded four distinct modes. The most distant mode (~20.3%–22.1%, mean = 21.2%) represented comparisons between *Ophiacantha* and *Ophionotus*, and second mode (~4.2%–5.3%, mean = 4.6%) represents comparisons between *O. victoriae* and *O. hexactis*. Finally, comparisons within *O. victoriae* samples yielded two distinct modes. The mode closest to the origin (~1.8%–4.0% mean = 2.8%) represents comparisons between individuals restricted to subnetwork lineages I, II, and III illustrated in Figure 3a, whereas the other mode (~0.2%–1.6%, mean = 0.5%) are comparisons of individuals between subnetwork lineages. Given that these subnetworks largely correspond to geographic regions and given results of the 2b‐RAD data (below), these latter two modes apparently correspond to intraspecific and interspecific variation, respectively.

### 3.2 2b‐RAD analyses

**Figure 4:**
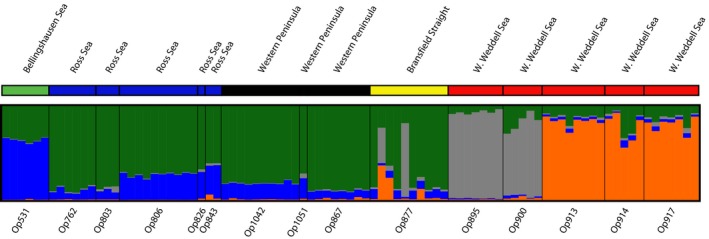
Patterns of population structure for *Ophionotus victoriae* based on SNP data analyzed in STRUCTURE 2.3.4. (Pritchard et al., 2000) and visualized in DISTRUCT (Rosenberg, 2004) testing for the true number of populations (*K*). *K* = 4 is presented in the graph above as our most likely accurate *K*

Following quality filtering and SNP calling, 16,588 loci were recovered (Table 3). To further filter these SNPs, any loci not present in at least 80% of samples were excluded, resulting in 1,999 remaining SNP loci. Next, any individuals with <80% of the total remaining SNP loci were excluded, resulting in removal of seven samples, thus leaving 89 individuals for analyses (Table 3). Under calculations of Delta *K* from STRUCTURE HARVESTER for this filtered and reduced dataset, *K* of 8 had the highest average support for Delta *K* after seven runs, although individual runs of *K* at 2 and 4 had the highest maximum‐likelihood scores (Figure S2). Thus, to assess which K was the most appropriate, STRUCTURE analyses were conducted with *K* set to 8, 4, and 2, then subjected to pairwise *F*_ST_ tests. At *K *=* *2 and 4, all populations were significantly different (Table 4) from one another, while a *K *=* *8 identified several populations with low nonsignificant *F*_ST_ values, signifying little to no structure between them and possibly an overestimation of *K*. As a result, *K *=* *4 was deemed the most appropriate for *O. victoriae*. Table S2 provides specific *F*_ST_ values between all 15 sampling localities used in 2b‐RAD SNP analyses. Results of the DISTRUCT graph from STRUCTURE are shown for *K *=* *4 in Figure 4 (Figures S3 and S4 depict *K *=* *2 and *K *=* *8, respectively).

**Table 3:** Filtering steps for 2b‐RAD SNP data

Filter	Samples	SNP Loci
All samples and SNP loci	96	16,588
Remove loci with <80% coverage	96	1,999
Remove samples with <80% SNP loci	89	1,999

**Table 4:** 2b‐RAD Pairwise *F*_ST_ values. Significance value (*p* < .05)

Region	(R/WP)	(B)	(WA/BS)	(WB)
Ross/Western Peninsula (R/WP)	**–**			
Bellingshausen (B)	0.12921	**–**		
Weddell/Bransfield Strait (WA/BS)	0.09789	0.10985	**–**	
Weddell (WB)	0.12676	0.13214	0.08039	**–**

**Figure 5:**
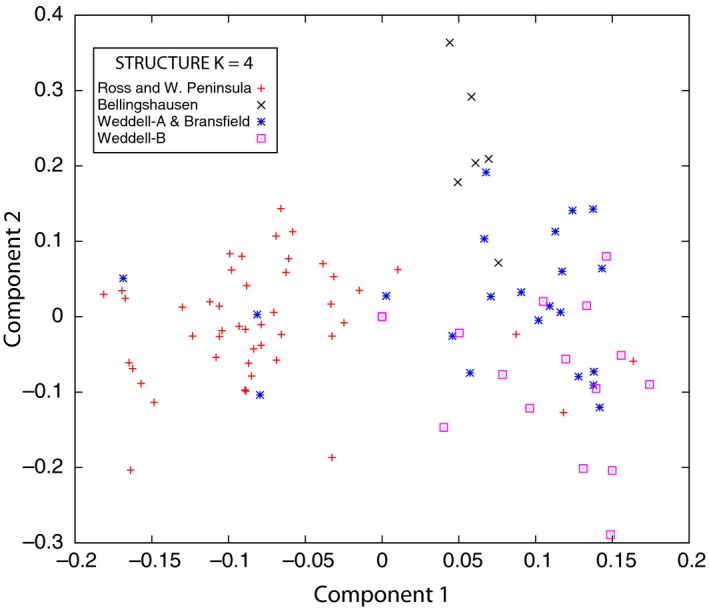
PCA results based on SNP data for samples labeled by the STRUCTURE's *K *=* *4 genetic populations. Weddell‐A/Bransfield population samples that intermix with the Ross Sea/Western Peninsula population were all from the sampling locality in the Bransfield Strait

Population structure was further investigated with SMARTPCA analyses that revealed geographic structuring in concordance with STRUCTURE. Figure 5 and Table S3 portray significant PCA results with samples labeled by genetic populations identified by STRUCTURE's *K *=* *4. All pairwise comparisons of the four STRUCTURE populations were significantly different as judged by a chi‐square test with a *p* < .01 cutoff (Table S3). To understand whether major geographic regions coincided with inferred STRUCTURE and SMARTPCA populations, we also pooled samples by geographic regions identified in methods (Figure S5 and Table S4). SMARTPCA results reveal significant differences between the Ross Sea/western Antarctic Peninsula, the Bellingshausen Sea, and the Weddell Sea. However, the Bransfield Strait appears to be an intermixing zone.

BayeScan analysis of filtered SNP loci reported only one locus under possible selection. When the sequence containing this SNP was searched using BLAST on the NCBI webserver, a 100% match came back to two different genes, specifically leucoanthocyanidin dioxygenase and fam206a. Due to the nature of the short 36‐base pair fragment, we cannot be positive as to the true identity of the SNP‐containing fragment. Furthermore, Hardy–Weinberg equilibrium was not violated for any loci (summary analyses for every locus in every population; a χ^2^ = 91.12, *df* = 108 and *p* = .879).

**Figure 6:**
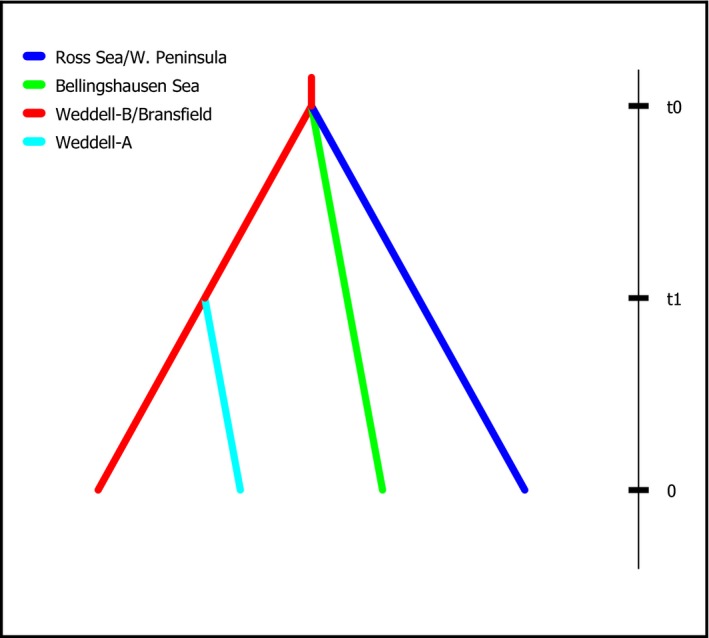
Highest supported scenarios using Bayesian computation (ABC). In these scenarios, *t*# represents time in generations and is based off the four genetic populations identified by STRUCTURE. All three geographic regions split at approximately the same time with a more recent diversification in the Weddell Sea

ABC analyses compared the fit of seven historical scenarios for the four genetic populations identified by STRUCTURE. These seven scenarios were chosen based on results of the mtDNA analyses, geographic history of Antarctica, and knowledge of oceanic currents. Each of the four populations includes two Weddell Sea populations, a Bellingshausen Sea population, and a population that includes both the western Antarctic Peninsula and the Ross Sea (consistent with Figure 4). In the highest scoring scenario, Scenario 1, the Bellingshausen, Weddell, and Ross/Western Peninsula populations separate at approximately the same time with a more recent diversification in the Weddell Sea (Figure 6; all scenarios presented in the Figure S1).

The combination of mtDNA COI sequence and nuclear SNP data provided strong evidence for regional genetic structure of *O. victoriae*. Based on analyses of total SNP data (STRUCTURE analyses, Figure 4 and pairwise *F*_ST_ Table 4), four genetically distinct populations are clearly identified: Ross Sea/Western Peninsula (R/WP), Bellingshausen Sea (B), Weddell Sea‐A/Bransfield Strait (WA/BS), and Weddell Sea‐B (WB) populations. Of the two populations within Weddell Sea, one population (WB) consists of individuals from two different mtDNA lineages (I and II) collected at three sampling localities between Seymour Island (a.k.a. Marambio Island) and the Antarctic Peninsula. The other (WA/BS) was recovered from two sampling localities south of Seymour Island and one sampling location in the Bransfield Strait (WA/BS). Population (B), which occurs between the two geographic regions comprising the (R/WP) population, was the most genetically differentiated with an average pairwise *F*_ST_ of (0.1237) among populations.

## 4 Discussion

Both mtDNA and 2b‐RAD data reveal considerable genetic structure across the Western Antarctic in the brittle star *O. victoriae*, questioning its current status as a single species. As mentioned, specimens were examined and morphological differences were not discernable with current taxonomy. However, both mtDNA and 2b‐RAD data suggest distinct genetic lineages within what is currently recognized as *O. victoriae*. Although the Bransfield Strait appears to be more diverse then other populations indicating a possible mixing zone, the degree of genetic structuring appears ordered by major geographic regions.

### 4.1 Phylogeographic patterns from mtDNA

Based on analyses of mtDNA COI, the western Weddell Sea has a recent shared history, or is currently connected with, the oceanic islands, and interestingly, the eastern Weddell Sea samples share a discrete haplotype subnetwork with the Ross Sea and Western Peninsula (Figure 3a, lineage III, 2b‐RAD data not available for eastern Weddell and oceanic islands). Lineage III, which includes the Ross Sea and eastern Weddell Sea samples, was also the most geographically widespread clade and yet the least variable, as no haplotypes are more than two steps from the most common haplotype. This particular lineage could represent support for the assumed circumpolar distribution of *O. victoriae* or at least large‐scale geographic dispersal capabilities through the Ross Gyre and out into the ACC.

Previous studies of other Antarctic benthic fauna have also revealed unexpected genetic structure in broadly distributed taxa. For example, the Antarctic crinoid *Promachocrinus kerguelensis* has a pelagic larval stage and was assumed to have a circumpolar distribution, but was ultimately found to be comprised of six different lineages and at least five different unrecognized species on the Antarctic Peninsula alone (Wilson, Hunter, Lockhart, & Halanych, 2007). Later analyses revealed all six lineages to be circumpolar, likely sympatric and eurybathic, with only two unrecognized species (Hemery et al., 2012). Similarly, population genetic analyses of two abundant and widespread SO pycnogonids, *Colossendeis megalonyx* and *Colossendeis robusta*, revealed multiple cryptic species as well (Dietz, Pieper, Seefeldt, & Leese, 2015; Krabbe, Leese, Mayer, Tollrian, & Held, 2010). Genetic studies (Held & Wägele, 2005; Hunter & Halanych, 2008; Janosik, Mahon, & Halanych, 2010; Leese, Kop, Wägele, & Held, 2008; Mahon, Thornhill, Norenburg, & Halanych, 2009; Mahon et al., 2008; Sands, O'Hara, Barnes, & Martín‐Ledo, 2015; Thornhill et al., 2008) revealed underestimation of species diversity in the SO and have shown multiple genetic lineages within a single morphologically defined species. Given the genetic structure our analyses recovered, unrecognized species may exist within *O. victoriae*, although no distinguishing morphological characteristics could be determined. In contrast, some species do appear to have a circumpolar distribution such as the Antarctic krill, *Euphausia superba*, which has a holopelagic life cycle. Specifically, Hofmann and Murphy's (2004) hypothesis that while individual adult krill may not circumnavigate the SO in their lifetime, slow continuous gene flow occurs, and this hypothesis was supported by recent, RAD‐tag analyses indicative of panmixia (Deagle, Faux, Kawaguchi, Meyer, & Jarman, 2015). With long‐lived pelagic larvae, similar dispersal capabilities are possible for *O. victoriae* as well and a 2b‐RAD‐based analysis is particularly appropriate.

### 4.2 Phylogeographic patterns from 2b‐RAD

Given that echinoderm larvae can remain in the water column for several months in the SO (Pearse et al., 1991), a circumpolar distribution for *O. victoriae* was a plausible hypothesis. Although we make the case that *O. victoriae* contains three distinct divergent mtDNA lineages, one lineage shows genetic connectivity over several thousands of kilometers. Specifically, 2b‐RAD data revealed the Ross Sea and most of the Western Peninsula individuals (excluding one sampling locality in the Bransfield Strait) to be a single genetic population. One likely reason for this observation is transportation of planktotrophic larvae by the ACC from the Ross Sea to the Western Peninsula. The ACC contacts the northern tip of the Western Peninsula. Depending on the depth planktotrophic larvae reside, the Circumpolar Deep Water and Upper Circumpolar Deep Water could have a significant impact on their distribution as well (Tynan, 1998).

The level of genetic differentiation recovered from 2b‐RAD between *O. victoriae* populations in both STRUCTURE and SMARTPCA analyses reveals distinct geographic structure. COI data further corroborate 2b‐RAD data in that *O. victoriae* in the Bellingshausen Sea and Amundsen Sea appear to represent a singular, largely disconnected clade (Figure 3a, lineage I). DIYABC analyses most strongly supported scenario 1 where the three geographic regions separated from each other early in their history with a secondary, more recent, diversification in the Weddell Sea. The isolation of the Amundsen and Bellingshausen Seas likely resulted from shifts in the position of the ACC provide a plausible explanation for the genetic differentiation recovered. Antarctic coastal currents likely are a factor in this isolation as they move the opposite direction of the ACC and have been used to explain genetic structure in benthic invertebrates thought to be circumpolar (Riesgo, Taboada, & Avila, 2015).

### 4.3 Overall structure

The high‐resolution 2b‐RAD approach was consistent with findings from COI while providing greater genetic resolution. Although 2b‐RAD data were more geographically and numerically limited relative to COI, both recovered strong connections between the Ross Sea and Western Peninsula, a distance of over 5,000 km, bypassing the Bellingshausen and Amundsen Seas. This connection is likely the result of transport from the Ross Gyre into the ACC, which does not contact the Antarctic shelf again until the western portion of the Antarctic Peninsula (Tynan, 1998). As seen in other taxa (e.g., *Promachocrinus kerguelensis*, Wilson et al., 2007; *Doris kerguelenensis* Wilson, Maschek, & Baker, 2013 *; Odontaster validus*, Janosik et al., 2010; *Nymphon australe*, Mahon et al., 2008), the northern tip of the Antarctic Peninsula, especially the Bransfield Strait, is an area of high genetic diversity. SMARTPCA analyses of SNP data provide additional support that the Bransfield Strait is a genetically diverse and likely an intermixing site for *O. victoriae* populations.

Probable causes for this diversity may be mixing of distinct water masses, including water from the ACC, and thus populations in the region (Gill, 1973; Smith, Hofmann, Klinck, & Lascara, 1999), repeated formation, and disintegration of refugia during glaciation events (Clarke & Crame, 1992) or other processes that promote mixing of populations. Although the ACC serves as a vector for eastward distribution, westward counter currents closer to the shelf and several large Antarctic gyres (including in the Weddell and Ross Seas) may further distribute, or isolate, populations (Thatje, 2012). For example, the Weddell Gyre moves clockwise and spills into the Bransfield Straits mixing with warmer waters (García et al., 2002; Kaiser et al., 2011). Such a situation supports our findings for isolation of the WA/BS population. Bransfield Strait consists of several water masses differing in oxygen and salinity (Gordon, Visbeck, & Huber, 2001) compared to those on the western Antarctic Peninsula, along with being hypothesized as an area of refugium during glaciation events (Jażdżewska, 2011). The separation of water masses and support for historical refugium both provide an explanation for genetic distinctiveness of the WA/BS population and R/WP population. Mitochondrial data suggest that the eastern Weddell Sea might share more similarities with the Ross Sea and Western Peninsula lineage than with the western Weddell Sea. Both types of data recovered a geographically structured distribution for *O. victoriae*. Whereas some lineages have very broad distributions and may be circumpolar, others are more restricted.

### 4.4 Comparing mtDNA to 2b‐RAD

Our study afforded the opportunity to compare traditional mtDNA markers to a whole‐genome SNP‐based approach such as 2b‐RAD. Other studies have recognized the ability of RAD data to recover structure that traditional markers have overlooked (Reitzel et al., 2013; Wagner et al., 2013). Although general phylogeographic structure of large‐scale SO regions was able to be ascertained through mtDNA, identification of four distinct populations and existence of population WB would have gone unrecognized if higher resolution 2b‐RAD analyses had not been employed. Population WB comprised 16 specimens whose haplotypes were within COI lineage I, with an additional four individuals from lineage II, which could have resulted from incomplete lineage sorting as mtDNA is uniparentally inherited. The 2b‐RAD data were able to reveal this higher resolution structure through fewer samples from a smaller geographic range. Further 2b‐RAD data in the sub‐Antarctic islands and the eastern Weddell Sea would provide greater insight into connectivity of organisms in the SO ecosystem with the Ross and Weddell Seas being of particular interest. As marine systems are often considered to have few barriers, these high‐resolution approaches provide us with better tools to answer ecological questions. With climate change prone to reshape current community structure in the SO ecosystem, large high‐resolution phylogeographic studies can help to serve as a benchmark or snapshot prior to any further restructuring.

## Conflict of Interest

None declared.

## Data Accessibility

All sequences collected herein are reported under GenBank accession numbers KY048203‐KY048268. Raw reads for 2b‐RAD SNP data are deposited to SRA SAMN05944630‐SAMN5944718. Data matrices and alignments are deposited in Dryad under accession doi:10.5061/dryad.0k1r0.
