# Supplementary material for: Geographic structure in the Southern Ocean circumpolar brittle star Ophionotus victoriae (Ophiuridae) revealed from mtDNA and single‐nucleotide polymorphism data
Source: Ecol Evol. 2016 Dec 16;7(2):475–85. doi: 10.1002/ece3.2617 (PMC5243193; doi:10.1002/ece3.2617)
Supplement: Supplementary file 1 [file ECE3-7-475-s001.docx]

**Supplementary Table 1.** Sampling information sorted by locality. Blank spaces represent data that was not available.

| **Sampling Group** | **Latitude** | **Longitude** | **# of 16S** | **# of COI** | **# of 2b-RAD** | **Year** | **Depth (m)** | **Cruise** | **Station** | **AMOVA**  **Region** |
| --- | --- | --- | --- | --- | --- | --- | --- | --- | --- | --- |
| 787 | -76.998275 | -175.0932 | 9 | 9 | 0 | 2013 | 541 | NBP-12-10 | 22 | Ross |
| 803 | -76.245261 | 174.50412 | 9 | 9 | 3 | 2013 | 604 | NBP-12-10 | 23 | Ross |
| 806 | -76.9038 | 169.96525 | 10 | 10 | 10 | 2013 | 764 | NBP-12-10 | 24 | Ross |
| 826 | -74.70781 | 168.40783 | 3 | 3 | 1 | 2013 | 489 | NBP-12-10 | 26 | Ross |
| 818 | -75.833465 | 166.50549 | 11 | 11 | 0 | 2013 | 552 | NBP-12-10 | 25 | Ross |
| 762 | -78.06324 | -169.99115 | 6 | 6 | 6 | 2013 | 549 | NBP-12-10 | 21 | Ross |
| 531 | -71.699 | -93.693667 | 9 | 9 | 6 | 2013 | 670 | NBP-12-10 | 3 | Bell/Amund |
| 843 | -74.995422 | 165.74422 | 6 | 6 | 2 | 2013 | 1101 | NBP-12-10 | 28 | Ross |
| 867 | -63.805542 | -60.479083 | 9 | 9 | 8 | 2013 | 428 | LMG-13-12 | 3 | W. Peninsula |
| 877 | -62.995875 | -58.598617 | 10 | 10 | 10 | 2013 | 320 | LMG-13-12 | 4 | W. Peninsula |
| 895 | -64.302283 | -56.136417 | 7 | 7 | 7 | 2013 | 290 | LMG-13-12 | 6 | Weddell |
| 900 | -64.303633 | -56.141533 | 0 | 0 | 5 | 2013 | 276 | LMG-13-12 | 6b | Weddell |
| 913 | -64.134392 | -56.860217 | 10 | 10 | 8 | 2013 | 310 | LMG-13-12 | 8 | Weddell |
| 914 | -63.742367 | -57.431867 | 8 | 8 | 5 | 2013 | 692 | LMG-13-12 | 9 | Weddell |
| 917 | -63.685783 | -56.859 | 10 | 10 | 7 | 2013 | 400 | LMG-13-12 | 10 | Weddell |
| 1051 | -64.64 | -64.245383 | 0 | 0 | 1 | 2013 | 695 | LMG-13-12 | 27 | W. Peninsula |
| 1042 | -64.846183 | -62.959483 | 10 | 10 | 10 | 2013 | 301 | LMG-13-12 | 26 | W. Peninsula |
| 57 | -63.666667 | -57.329167 | 8 | 8 | 0 | 2004 | 335 | LMG-04-14 | 40 | Weddell |
| 59 | -63.666667 | -57.329167 | 3 | 3 | 0 | 2004 | 335 | LMG-04-14 | 40 | Weddell |
| 312 | -64.350361 | -61.759953 | 8 | 8 | 0 | 2006 | 334 | LMG-06-05 | 17 | W. Peninsula |
| 398 | -67.717028 | -68.243286 | 7 | 7 | 0 | 2006 | 170 | LMG-06-05 | 47 | W. Peninsula |
| 422 | -65.183694 | -64.243283 | 10 | 10 | 0 | 2006 | 285 | LMG-06-05 | 58 | W. Peninsula |
| 82 | -62.850361 | -59.45995 | 5 | 5 | 0 | 2004 | 900 | LMG-04-14 | 47 | W. Peninsula |
| 92 | -63.383694 | -60.05995 | 15 | 15 | 0 | 2004 | 277 | LMG-04-14 | 51 | W. Peninsula |
| 321 | -64.350361 | -57.076617 | 5 | 5 | 0 | 2006 | 146 | LMG-06-05 | 21 | Weddell |
| 73 | -62.100361 | -58.393283 | 10 | 10 | 0 | 2004 | 276 | LMG-04-14 | 44 | W. Peninsula |
| 362 | -67.733694 | -69.293283 | 10 | 10 | 0 | 2006 | 122 | LMG-06-05 | 33 | W. Peninsula |
| 114 | -62.933694 | -60.65995 | 10 | 10 | 0 | 2004 | 161 | LMG-04-14 | 64 | W. Peninsula |
| 194 | -58.783694 | -26.343283 | 9 | 9 | 0 | 200R | 270 | LMG-04-14 | 34 | Oceanic islands |
| 195 | -57.088533 | -30.398971 | 6 | 6 | 0 | 2006 | 130 | LMG-04-14 | 32 | Oceanic islands |
| 196 | -56.00683 | 2.6013889 | 8 | 8 | 0 | 2006 | 648 | LMG-04-14 | 50 | Oceanic islands |
| 177 | -54.816665 | -3.5 | 10 | 10 | 0 | 2004 | 169 | LMG-04-14 | 58 | Oceanic islands |
| AGT-21A | -67.546004 | -70.189001 | 0 | 1 | 0 | 2009 | 507 | JR230 | N/A | W. Peninsula |
| EI-AGT-4 | -61.334004 | -55.195 | 0 | 9 | 0 | 2006 | 201 | JR144 | N/A | W. Peninsula |
| EI-AGT-3 | -61.385998 | -55.192999 | 0 | 5 | 0 | 2006 | 482 | JR144 | N/A | W. Peninsula |
| PB-AGT-1B | -61.035998 | -46.955 | 0 | 1 | 0 | 2006 | 1630 | JR144 | N/A | Oceanic islands |
| ST-AGT-3 | -59.481 | -27.278999 | 0 | 2 | 0 | 2006 | 549 | JR144 | N/A | Oceanic islands |
| ST-EBS-4 | -59.47 | -27.276001 | 0 | 1 | 0 | 2006 | 307 | JR144 | N/A | Oceanic islands |
| ST-AGT-1 | -59.518003 | -27.436 | 0 | 9 | 0 | 2006 | 1545 | JR144 | N/A | Oceanic islands |
| LI-AGT-4 | -62.525 | -61.826997 | 0 | 2 | 0 | 2006 | 192 | JR144 | N/A | W. Peninsula |
| RGBT-02 | -61.965998 | -57.244002 | 0 | 1 | 0 | 2006 | 129 | JR144 | N/A | W. Peninsula |
| LI-AGT-1 | -62.276 | -61.595999 | 0 | 4 | 0 | 2006 | 1511 | JR144 | N/A | W. Peninsula |
| BIO6-AGT-2B | -71.179002 | -109.894 | 0 | 19 | 0 | 2008 | 998 | JR179 | N/A | Bell/Amund |
| BIO6-AGT-2A | -71.175003 | -109.863 | 0 | 22 | 0 | 2008 | 1079 | JR179 | N/A | Bell/Amund |
| BIO6-AGT-2C | -71.182 | -109.926 | 0 | 15 | 0 | 2008 | 986 | JR179 | N/A | Bell/Amund |
| BIO6-AGT-1B | -71.152001 | -110.013 | 0 | 1 | 0 | 2008 | 1491 | JR179 | N/A | Bell/Amund |
| BIO6-AGT-1A | -71.146001 | -109.971 | 0 | 1 | 0 | 2008 | 1530 | JR179 | N/A | Bell/Amund |
| PS77-252-3 | -64.694001 | -60.517999 | 0 | 1 | 0 | 2011 | 316 | PS77 | N/A | Weddell |
| PS77-248-3 | -65.924 | -60.332 | 0 | 1 | 0 | 2011 | 433 | PS77 | N/A | Weddell |
| PS77-250-6 | -65.383999 | -61.548001 | 0 | 5 | 0 | 2011 | 566 | PS77 | N/A | Weddell |
| PS77-235-8 | -65.528002 | -61.551999 | 0 | 1 | 0 | 2011 | 448 | PS77 | N/A | Weddell |
| PS77-237-2 | -66.209002 | -60.162001 | 0 | 1 | 0 | 2011 | 382 | PS77 | N/A | Weddell |
| PS77-265-2 | -70.794 | -10.67 | 0 | 4 | 0 | 2011 | 633 | PS77 | N/A | Weddell |
| PS77-260-6 | -70.84 | -10.597 | 0 | 2 | 0 | 2011 | 259 | PS77 | N/A | Weddell |
| PS77-312-2 | -54.47 | 3.1849998 | 0 | 5 | 0 | 2011 | 297 | PS77 | N/A | Oceanic islands |
| PS77-291-1 | -70.841998 | -10.587 | 0 | 3 | 0 | 2011 | 267 | PS77 | N/A | Weddell |
| PS77-312-4 | -54.481 | 3.1889998 | 0 | 4 | 0 | 2011 | 300 | PS77 | N/A | Oceanic islands |
| PS77-312-3 | -54.502001 | 3.2249999 | 0 | 4 | 0 | 2011 | 264 | PS77 | N/A | Oceanic islands |
| PS77-308-1 | -70.854999 | -10.589001 | 0 | 1 | 0 | 2011 | 223 | PS77 | N/A | Weddell |
| PS77-301-1 | -70.850999 | -10.588001 | 0 | 1 | 0 | 2011 | 225 | PS77 | N/A | Weddell |
| PS77-284-1 | -70.972 | -10.504002 | 0 | 1 | 0 | 2011 | 289 | PS77 | N/A | Weddell |
| PS77-275-3 | -70.934 | -10.496 | 0 | 1 | 0 | 2011 | 238 | PS77 | N/A | Weddell |
| PS77-222-5 | -62.297002 | -58.678 | 0 | 1 | 0 | 2011 | 873 | PS77 | N/A | W. Peninsula |
| PS77-239-3 | -66.195001 | -60.148998 | 0 | 5 | 0 | 2011 | 362 | PS77 | N/A | Weddell |
| PS77-226-7 | -64.914001 | -60.620998 | 0 | 5 | 0 | 2011 | 226 | PS77 | N/A | Weddell |
| PS77-228-3 | -64.918001 | -60.537 | 0 | 5 | 0 | 2011 | 279 | PS77 | N/A | Weddell |
| PS77-233-3 | -65.557999 | -61.621998 | 0 | 5 | 0 | 2011 | 324 | PS77 | N/A | Weddell |
| PS77-228-4 | -64.929 | -60.564999 | 0 | 1 | 0 | 2011 | 315 | PS77 | N/A | Weddell |
| AGT-2B | -67.983001 | -68.438 | 0 | 1 | 0 | 2009 | 585 | JR230 | N/A | W. Peninsula |
| BIO5-AGT-3C | -73.986 | -107.39 | 0 | 1 | 0 | 2008 | 541 | JR179 | N/A | Bell/Amund |
| BIO4-AGT-2C | -74.477 | -104.257 | 0 | 5 | 0 | 2008 | 1150 | JR179 | N/A | Bell/Amund |

**Supplementary Table 2.** F_ST_ values for 2b-RAD data based on sampling locality. Significant values (P < 0.05).

| Fst P values | 1042 | 1051 | 531 | 762 | 803 | 806 | 826 | 843 | 867 | 877 | 895 | 900 | 913 | 914 |
| --- | --- | --- | --- | --- | --- | --- | --- | --- | --- | --- | --- | --- | --- | --- |
| 1042 | - |  |  |  |  |  |  |  |  |  |  |  |  |  |
| 1051 | 0.0715 | - |  |  |  |  |  |  |  |  |  |  |  |  |
| 531 | 0.0880 | 0.1606 | - |  |  |  |  |  |  |  |  |  |  |  |
| 762 | 0.0516 | 0.1385 | 0.1173 | - |  |  |  |  |  |  |  |  |  |  |
| 803 | 0.0597 | 0.1926 | 0.1341 | 0.0883 | - |  |  |  |  |  |  |  |  |  |
| 806 | 0.0426 | 0.0784 | 0.0905 | 0.0484 | 0.0630 | - |  |  |  |  |  |  |  |  |
| 826 | 0.0784 | 0.4402 | 0.1694 | 0.1204 | 0.2034 | 0.0773 | - |  |  |  |  |  |  |  |
| 843 | 0.0708 | 0.2972 | 0.1433 | 0.1041 | 0.1584 | 0.0652 | 0.3118 | - |  |  |  |  |  |  |
| 867 | 0.0430 | 0.0962 | 0.0956 | 0.0605 | 0.0735 | 0.0480 | 0.0966 | 0.0865 | - |  |  |  |  |  |
| 877 | 0.0427 | 0.0708 | 0.0807 | 0.0609 | 0.0620 | 0.0515 | 0.0725 | 0.0651 | 0.0445 | - |  |  |  |  |
| 895 | 0.0817 | 0.1382 | 0.1102 | 0.1019 | 0.1113 | 0.0890 | 0.1254 | 0.1269 | 0.0861 | 0.0555 | - |  |  |  |
| 900 | 0.0871 | 0.1705 | 0.1235 | 0.1157 | 0.1249 | 0.0931 | 0.1461 | 0.1511 | 0.0950 | 0.0584 | 0.0639 | - |  |  |
| 913 | 0.0987 | 0.1478 | 0.1223 | 0.1290 | 0.1339 | 0.1114 | 0.1422 | 0.13532 | 0.1085 | 0.0680 | 0.0746 | 0.0890 | - |  |
| 914 | 0.1095 | 0.1974 | 0.1492 | 0.1400 | 0.1613 | 0.1180 | 0.2008 | 0.1854 | 0.1183 | 0.0718 | 0.0876 | 0.1121 | 0.0664 | - |
| 917 | 0.1019 | 0.1584 | 0.1317 | 0.1295 | 0.1326 | 0.1170 | 0.1534 | 0.1426 | 0.1109 | 0.0673 | 0.0751 | 0.0957 | 0.0506 | 0.0735 |

**Supplementary Table 3.** PCA χ^2^ results for genetic populations identified by STRUCTURE K=4. Significant values (P<0.01)* and (P<0.001)** are in bold.

| *χ^2^* | Ross/W. Peninsula | Bellingshausen | Weddell A/Bransfield | Weddell B |
| --- | --- | --- | --- | --- |
| Ross/W. Peninsula | - | **-** | **-** | **-** |
| Bellingshausen | **66.451**** | - | **-** | **-** |
| Weddell A/Bransfield | **54.004**** | **35.626**** | - | - |
| Weddell B | **60.923**** | **42.120**** | **25.034*** | - |

**Supplementary Table 4.** PCA χ^2^ results for samples labeled by geographic region. Significant values (P<0.01)* and (P<0.001)** are in bold.

| *χ^2^* | Ross Sea | Bellingshausen Sea | Western Peninsula | Bransfield Strait | Weddell Sea |
| --- | --- | --- | --- | --- | --- |
| Ross Sea | - | **-** | - | - | **-** |
| Bellingshausen Sea | **80.403**** | - | **-** | **-** | **-** |
| Western Peninsula | 17.080 | **84.254**** | - | - | **-** |
| Bransfield Strait | **27.269*** | **32.062**** | 12.454 | - | - |
| Weddell Sea | **96.670**** | **42.525**** | **56.393**** | 20.925 | - |

**Supplementary Table 5.** Analysis of molecular variance statistics for O. victoriae based on 16S data.

| Source of variation | d.f. | Sum of squares | σ^2^ | Percentage of variation |
| --- | --- | --- | --- | --- |
| Among groups | 4 | 270.700 | 0.780 | 22.95361 |
| Among populations within groups | 3 | 81.044 | 0.946 | 27.81462 |
| Within populations | 243 | 406.744 | 1.674 | 49.23177 |
| Total | 250 | 758.518 | 3.400 |  |

**Supplementary Table 6.** Analysis of molecular variance statistics for O. victoriae based on COI & 16S data.

| Source of variation | d.f. | Sum of squares | σ^2^ | Percentage of variation |
| --- | --- | --- | --- | --- |
| Among groups | 4 | 8997.445 | 19.178 | 19.76305 |
| Among populations within groups | 3 | 3444.274 | 41.491 | 42.75669 |
| Within populations | 243 | 8838.085 | 36.371 | 37.48025 |
| Total | 250 | 21279.805 | 97.040 |  |

**Supplementary Table 7.** Summary statistics for DIYABC v2.1.0 historical scenario analyses. Values indicate for each summary statistics the proportion of simulated data sets which have a value below the observed one. A total of 7,000,000 simulated datasets were performed.

| Summary statistics | observed | scenario 1 | scenario 2 | scenario 3 | scenario 4 | scenario 5 | scenario 6 | scenario 7 |
| --- | --- | --- | --- | --- | --- | --- | --- | --- |
| FM1_1_1&2 | -0.1697 | 0.0373 (*) | 0.0001(***) | 0.0430 (*) | 0.0021 (**) | 0.0002(***) | 0.0843 | 0.0027(**) |
| FM1_1_1&3 | -0.0956 | 0.1208 | 0.2014 | 0.1263 | 0.1204 | 0.004(**) | 0.0212(*) | 0.2312 |
| FM1_1_1&4 | -0.1632 | 0.1739 | 0.3219 | 0.329 | 0.1741 | 0.0517 | 0.1159 | 0.5176 |
| FM1_1_2&3 | -0.1725 | 0.0788 | 0.0011(**) | 0.0792 | 0.0795 | 0.1183 | 0.0079(**) | 0.0073(**) |
| FM1_1_2&4 | -0.2344 | 0.0927 | 0.0249(*) | 0.2831 | 0.2835 | 0.2077 | 0.0827 | 0.0828 |
| FM1_1_3&4 | -0.1145 | 0.1748 | 0.2845 | 0.2065 | 0.1756 | 0.284 | 0.333 | 0.3363 |
| FV1_1_1&2 | -0.0344 | 0.0865 | 0.0013(**) | 0.0839 | 0.0095 (**) | 0.0014(**) | 0.1586 | 0.0101(*) |
| FV1_1_1&3 | -0.0128 | 0.1144 | 0.1876 | 0.1173 | 0.1141 | 0.0036(**) | 0.0192(*) | 0.2153 |
| FV1_1_1&4 | -0.033 | 0.1232 | 0.2619 | 0.2964 | 0.1229 | 0.0412(*) | 0.097 | 0.4748 |
| FV1_1_2&3 | -0.0366 | 0.1632 | 0.0095(**) | 0.1783 | 0.164 | 0.2468 | 0.0352(*) | 0.0354(*) |
| FV1_1_2&4 | -0.0535 | 0.1479 | 0.0586 | 0.3617 | 0.3616 | 0.2946 | 0.1256 | 0.147 |
| FV1_1_3&4 | -0.0188 | 0.1642 | 0.2653 | 0.1944 | 0.1648 | 0.265 | 0.314 | 0.3249 |
| FMO_1_1&2 | -0.0953 | 0.3585 | 0.1321 | 0.3068 | 0.1262 | 0.0284(*) | 0.5871 | 0.063 |
| FMO_1_1&3 | -0.0541 | 0.1823 | 0.3146 | 0.1287 | 0.1958 | 0.0029(**) | 0.0172(*) | 0.2962 |
| FMO_1_1&4 | -0.1055 | 0.3069 | 0.5676 | 0.5555 | 0.3054 | 0.1526 | 0.2445 | 0.7857 |
| FMO_1_2&3 | -0.0948 | 0.4465 | 0.1976 | 0.4493 | 0.4418 | 0.6734 | 0.2164 | 0.1408 |
| FMO_1_2&4 | -0.1346 | 0.6428 | 0.5181 | 0.7985 | 0.7754 | 0.832 | 0.5877 | 0.5562 |
| FMO_1_3&4 | -0.0587 | 0.24 | 0.3915 | 0.2036 | 0.2267 | 0.3888 | 0.4364 | 0.3878 |
| NM1_1_1&2 | -0.083 | 0.4113 | 0.1002 | 0.343 | 0.224 | 0.0675 | 0.5258 | 0.1583 |
| NM1_1_1&3 | -0.035 | 0.1654 | 0.2874 | 0.1583 | 0.1651 | 0.0085(**) | 0.0342(*) | 0.2812 |
| NM1_1_1&4 | -0.0715 | 0.2666 | 0.4215 | 0.4097 | 0.2673 | 0.1026 | 0.1802 | 0.5975 |
| NM1_1_2&3 | -0.084 | 0.292 | 0.0808 | 0.3621 | 0.2923 | 0.4583 | 0.1778 | 0.1536 |
| NM1_1_2&4 | -0.1198 | 0.3517 | 0.219 | 0.5184 | 0.5183 | 0.4989 | 0.311 | 0.3373 |
| NM1_1_3&4 | -0.0477 | 0.1994 | 0.3389 | 0.2575 | 0.2001 | 0.3385 | 0.3856 | 0.3901 |
| NV1_1_1&2 | -0.0223 | 0.3108 | 0.0323 | 0.2584 | 0.1082 | 0.0309(*) | 0.4229 | 0.0922 |
| NV1_1_1&3 | -0.0058 | 0.1235 | 0.2105 | 0.1218 | 0.1233 | 0.0044(**) | 0.0213(*) | 0.2227 |
| NV1_1_1&4 | -0.017 | 0.1142 | 0.2507 | 0.2725 | 0.1141 | 0.0339(*) | 0.084 | 0.4429 |
| NV1_1_2&3 | -0.0226 | 0.2362 | 0.0331 | 0.2857 | 0.2365 | 0.3749 | 0.0911 | 0.0848 |
| NV1_1_2&4 | -0.038 | 0.2141 | 0.0975 | 0.4271 | 0.4272 | 0.376 | 0.1829 | 0.2073 |
| NV1_1_3&4 | -0.0103 | 0.1671 | 0.2785 | 0.2065 | 0.1678 | 0.2784 | 0.3266 | 0.3366 |
| NMO_1_1&2 | -0.0811 | 0.7052 | 0.458 | 0.7795 | 0.5542 | 0.4575 | 0.9014 | 0.5234 |
| NMO_1_1&3 | -0.0348 | 0.2912 | 0.485 | 0.2663 | 0.2984 | 0.028(*) | 0.0752 | 0.4799 |
| NMO_1_1&4 | -0.0709 | 0.486 | 0.6799 | 0.7097 | 0.4882 | 0.3776 | 0.456 | 0.8504 |
| NMO_1_2&3 | -0.0828 | 0.7679 | 0.512 | 0.7284 | 0.7654 | 0.8823 | 0.5502 | 0.5036 |
| NMO_1_2&4 | -0.1145 | 0.8138 | 0.7435 | 0.9053 | 0.8857 | 0.93 | 0.7749 | 0.7788 |
| NMO_1_3&4 | -0.0467 | 0.4107 | 0.6151 | 0.4236 | 0.413 | 0.6226 | 0.6698 | 0.6017 |

**Supplementary Table 8. Genbank accession numbers for each haplotype and corresponding sequences.**

| Genbank accession COI | Sequence |
| --- | --- |
| KY048218 | DSOPH1899 |
|  | DSOPH1900 |
|  | Op913_3E_3 |
| FJ917329 | 194.1E.02 |
|  | 195.1E.05 |
|  | DSOPH1903 |
|  | DSOPH2142 |
|  | DSOPH2143 |
|  | DSOPH2144 |
|  | DSOPH2148 |
|  | DSOPH2152 |
| KY048223 | DSOPH2157 |
|  | DSOPH2159 |
| KY048231 | DSOPH2198 |
|  | DSOPH2212 |
|  | DSOPH2251 |
|  | DSOPH2678 |
|  | DSOPH2685 |
|  | DSOPH2734 |
|  | Op913_3E_10 |
| KY048226 | DSOPH2186 |
|  | DSOPH2215 |
|  | DSOPH2217 |
|  | DSOPH2254 |
|  | DSOPH2256 |
|  | DSOPH2258 |
|  | DSOPH2684 |
|  | DSOPH2699 |
|  | DSOPH2743 |
|  | Op531_3E_6 |
| KY048234 | DSOPH2203 |
|  | DSOPH2207 |
|  | DSOPH2218 |
|  | Op803_4C_1 |
|  | Op806_3C_2 |
| KY048229 | DSOPH2193 |
|  | DSOPH2206 |
|  | DSOPH2208 |
|  | DSOPH2211 |
|  | DSOPH2216 |
|  | DSOPH2230 |
|  | DSOPH2252 |
|  | DSOPH2259 |
|  | DSOPH2272 |
|  | DSOPH2676 |
|  | DSOPH2738 |
| KY048233 | DSOPH2201 |
|  | DSOPH2202 |
|  | DSOPH2209 |
|  | DSOPH2731 |
|  | DSOPH2752 |
| KY048228 | DSOPH2191 |
|  | DSOPH2199 |
|  | DSOPH2229 |
|  | DSOPH2255 |
|  | DSOPH2273 |
|  | DSOPH2733 |
|  | DSOPH2736 |
|  | DSOPH2903 |
|  | Op531_3E_13 |
|  | Op531_3E_9 |
| KY048232 | DSOPH2200 |
|  | DSOPH2276 |
| FJ917310 | 321.2C.04 |
|  | 57.3C.05 |
|  | 57.3C.10 |
|  | 57.3C.12 |
|  | 59.2C.03 |
|  | 59.2C.06 |
|  | 59.2C.07 |
|  | DSOPH1912 |
|  | DSOPH2161 |
|  | DSOPH2204 |
|  | DSOPH2888 |
|  | DSOPH3835 |
|  | Op913_3E_1 |
|  | Op913_3E_2 |
|  | Op913_3E_4 |
|  | Op913_3E_5 |
|  | Op913_3E_6 |
|  | Op913_3E_9 |
|  | Op914_3E_8 |
|  | Op914_3E_9 |
|  | Op917_3E_10 |
|  | Op917_3E_4 |
|  | Op917_3E_6 |
|  | Op917_3E_7 |
| KY048265 | Op914_3E_3 |
|  | Op914_3E_5 |
| KY048261 | Op762_3C_2 |
|  | Op787_6C_4 |
|  | Op806_3C_1 |
|  | Op806_7C |
|  | Op806_8C_1 |
|  | Op818_2E |
|  | Op818_3C_3 |
|  | Op818_4C_3 |
|  | Op818_4C_4 |
|  | Op826_3C_2 |
|  | Op843_3C_1 |
|  | Op843_7C_1 |
|  | Op843_7C_4 |
| FJ917348 | 422.1C.10 |
|  | DSOPH2568 |
|  | DSOPH2964 |
| FJ917339 | 312.3C.07 |
|  | 312.3C.09 |
|  | 422.1C.01 |
|  | 422.1C.04 |
|  | 422.1C.05 |
|  | 422.1C.08 |
|  | 422.1C.14 |
|  | E82.2C.01 |
|  | E82.2C.02 |
|  | DSOPH2327 |
|  | DSOPH724 |
|  | Op1042_3E_2 |
|  | Op1042_3E_3 |
|  | Op1042_3E_4 |
|  | Op1042_3E_9 |
|  | Op867_4E_10 |
|  | Op867_4E_2 |
|  | Op867_4E_3 |
|  | Op867_4E_6 |
|  | Op867_4E_8 |
|  | Op867_4E_9 |
|  | Op877_2E_1 |
|  | Op877_2E_8 |
| FJ917337 | 312.3C.01 |
|  | 312.3C.03 |
|  | 312.3C.16 |
|  | 398.1E.12 |
|  | 422.1C.03 |
|  | 422.1C.06 |
|  | E82.2C.04 |
|  | E82.2C.05 |
|  | DSOPH1908 |
|  | DSOPH2154 |
|  | DSOPH2346 |
|  | DSOPH2571 |
|  | DSOPH2904 |
|  | DSOPH2962 |
|  | DSOPH2963 |
|  | DSOPH3033 |
|  | DSOPH3035 |
|  | DSOPH3185 |
|  | DSOPH3216 |
|  | DSOPH3239 |
|  | DSOPH3807 |
|  | DSOPH3810 |
|  | DSOPH3859 |
|  | DSOPH3871 |
|  | DSOPH446 |
|  | DSOPH721 |
|  | DSOPH722 |
|  | DSOPH725 |
|  | Op1042_3E_1 |
|  | Op1042_3E_6 |
|  | Op531_3E_11 |
|  | Op762_2E |
|  | Op762_5C_1 |
|  | Op762_6C_1 |
|  | Op762_6C_2 |
|  | Op787_5C_1 |
|  | Op787_5C_2 |
|  | Op787_5C_3 |
|  | Op787_5C_4 |
|  | Op787_6C_1 |
|  | Op787_6C_2 |
|  | Op787_6C_3 |
|  | Op787_6C_5 |
|  | Op803_3C_1 |
|  | Op803_3C_2 |
|  | Op803_3C_3 |
|  | Op803_3C_4 |
|  | Op803_3C_5 |
|  | Op803_3C_6 |
|  | Op803_4C_2 |
|  | Op803_4C_3 |
|  | Op806_2E |
|  | Op806_3C_3 |
|  | Op806_3C_4 |
|  | Op806_8C_2 |
|  | Op806_8C_3 |
|  | Op806_8C_4 |
|  | Op818_3C_1 |
|  | Op818_3C_2 |
|  | Op818_3C_4 |
|  | Op818_3C_5 |
|  | Op818_4C_1 |
|  | Op818_4C_2 |
|  | Op818_4C_5 |
|  | Op826_2E |
|  | Op826_3C_1 |
|  | Op843_2E |
|  | Op843_7C_2 |
|  | Op843_7C_3 |
|  | Op867_4E_5 |
|  | Op867_4E_7 |
|  | Op877_2E_3 |
|  | Op877_2E_7 |
|  | Op877_2E_9 |
| FJ917340 | 312.3C.15 |
|  | DSOPH1756 |
|  | DSOPH2155 |
|  | DSOPH2971 |
|  | DSOPH3098 |
|  | Op1042_3E_5 |
|  | Op867_4E_1 |
|  | Op877_2E_5 |
| FJ917333 | 194.1E.07 |
|  | 195.1E.04 |
| FJ917328 | 194.1E.01 |
|  | 194.1E.06 |
|  | 195.1E.03 |
|  | 195.1E.06 |
|  | 195.1E.08 |
|  | DSOPH1904 |
|  | DSOPH2149 |
| FJ917332 | 194.1E.05 |
|  | 195.1E.07 |
|  | DSOPH2145 |
| FJ917324 | 177.1E.01 |
|  | 177.1E.05 |
|  | 196.1E.07 |
|  | DSOPH3146 |
| FJ917326 | 177.1E.04 |
|  | 177.1E.07 |
|  | 177.1E.10 |
|  | 177.1E.12 |
|  | 196.1E.01 |
|  | 196.1E.04 |
|  | 196.1E.05 |
|  | 196.1E.06 |
|  | 196.1E.08 |
|  | DSOPH3043 |
|  | DSOPH3045 |
|  | DSOPH3046 |
|  | DSOPH3145 |
|  | DSOPH3147 |
|  | DSOPH3157 |
|  | DSOPH3160 |
| FJ917313 | 196.1E.10 |
|  | 57.3C.13 |
|  | 92.10C |
|  | Op913_3E_7 |
|  | Op917_3E_5 |
|  | Op917_3E_8 |
|  | Op917_3E_9 |
| FJ917312 | 114.5C |
|  | 57.3C.11 |
|  | DSOPH1909 |
|  | Op914_3E_10 |
| KY048252 | DSOPH3528 |
|  | DSOPH3873 |
|  | Op1042_3E_7 |
| KY048243 | DSOPH2914 |
|  | DSOPH3872 |
| KY048242 | DSOPH2902 |
|  | DSOPH2905 |
|  | DSOPH2906 |
| KY048239 | DSOPH2729 |
|  | Op531_3E_4 |
|  | Op531_3E_8 |
| KY048230 | DSOPH2195 |
|  | DSOPH2213 |
|  | DSOPH2253 |
|  | DSOPH2260 |
|  | DSOPH2264 |
|  | DSOPH2359 |
|  | DSOPH2680 |
|  | DSOPH2730 |
|  | DSOPH2732 |
|  | DSOPH2747 |
|  | DSOPH2753 |
|  | DSOPH678 |
| KY048246 | DSOPH3096 |
|  | DSOPH3311 |
| FJ917327 | 177.1E.02 |
|  | 177.1E.06 |
|  | 177.1E.09 |
|  | 177.1E.11 |
|  | DSOPH3144 |
| FJ917316 | 114.10C |
|  | 114.4C |
|  | 114.7C |
|  | 114.8C |
|  | 362.1C.09 |
|  | 362.1C.12 |
|  | 398.1E.02 |
|  | 398.1E.14 |
|  | 92.11C |
|  | 92.13C |
|  | 92.15C |
|  | 92.17C |
|  | 92.5C |
|  | 92.6C |
|  | E73.2C.09 |
|  | DSOPH3848 |
|  | DSOPH3892 |
|  | Op877_2E_2 |
| FJ917322 | 114.13C |
|  | 114.6C |
|  | 312.3C.02 |
|  | 398.1E.07 |
|  | E73.2C.06 |
|  | DSOPH1910 |
|  | DSOPH2918 |
|  | DSOPH3809 |
|  | DSOPH3876 |
| FJ917319 | 321.2C.03 |
|  | 321.2C.06 |
|  | 92.12C |
|  | E73.2C.08 |
|  | DSOPH1898 |
|  | DSOPH2257 |
|  | Op895_3E_2 |
|  | Op895_3E_4 |
|  | Op895_3E_8 |
|  | Op913_3E_8 |
|  | Op914_3E_2 |
| FJ917311 | 57.3C.08 |
|  | Op917_3E_2 |
| FJ917343 | 362.1C.07 |
|  | 398.1E.01 |
|  | 398.1E.15 |
| FJ917320 | 114.3C |
|  | 92.16C |
|  | E73.2C.10 |
|  | DSOPH2924 |
|  | DSOPH3837 |
|  | DSOPH3839 |
|  | Op877_2E_10 |
|  | Op877_2E_4 |
|  | Op877_2E_6 |
| FJ917342 | 362.1C.01 |
|  | 362.1C.10 |
| FJ917318 | 321.2C.01 |
|  | 92.8C |
|  | E73.2C.02 |
|  | E73.2C.11 |
|  | DSOPH3808 |
|  | DSOPH3847 |
|  | DSOPH3850 |
|  | DSOPH3851 |
|  | Op914_3E_6 |
|  | Op914_3E_7 |
| FJ917309 | 362.1C.02 |
|  | 362.1C.03 |
|  | 362.1C.04 |
|  | 362.1C.05 |
|  | 57.3C.03 |
|  | 57.3C.14 |
|  | 92.14C |
|  | 92.9C |
|  | E73.2C.01 |
|  | E73.2C.05 |
|  | DSOPH1263 |
|  | DSOPH2156 |
|  | Op895_3E_5 |
|  | Op895_3E_7 |
| KY048241 | DSOPH2867 |
| FJ917347 | 422.1C.07 |
| KY048262 | Op762_4C_1 |
| FJ917346 | 422.1C.02 |
| KY048256 | Op1042_3E_10 |
| FJ917338 | 312.3C.05 |
| FJ917345 | 398.1E.13 |
| KY048257 | Op1042_3E_8 |
| KY048248 | DSOPH3120 |
| KY048247 | DSOPH3097 |
| KY048245 | DSOPH3044 |
| KY048235 | DSOPH2205 |
| KY048260 | Op531_3E_5 |
| KY048237 | DSOPH2566 |
| KY048236 | DSOPH2275 |
| KY048240 | DSOPH2742 |
| KY048221 | DSOPH2150 |
| KY048259 | OP531_3E_12 |
| KY048238 | DSOPH2567 |
| KY048227 | DSOPH2187 |
| KY048258 | Op531_3E_10 |
| KY048225 | DSOPH2160 |
| KY048249 | DSOPH3158 |
| FJ917317 | 92.7C |
| FJ917344 | 362.1C.11 |
| KY048254 | DSOPH3838 |
| KY048255 | DOPH3849 |
| KY048268 | DSOPH3811 |
| KY048253 | DSOPH3836 |
| FJ917353 | E73.2C.12 |
| KY048222 | DSOPH2151 |
| FJ917331 | 194.1E.04 |
| KY048264 | Op895_3E_3 |
| FJ917314 | 92.3C |
| KY048220 | DSOPH2146 |
| KY048219 | DSOPH1911 |
| FJ917330 | 194.1E.03 |
| FJ917354 | E82.2C.03 |
| KY048267 | Op917_3E_3 |
| KY048263 | Op895_3E_10 |
| FJ917321 | 114.2C |
| FJ917351 | E73.2C.03 |
| FJ917315 | 92.4C |
| FJ917323 | 114.11C |
| KY048251 | DSOPH3226 |
| FJ917341 | 321.2C.02 |
| KY048266 | Op917_3E_1 |
| FJ917335 | 194.1E.09 |
| FJ917334 | 194.1E.08 |
| KY048244 | DSOPH2923 |
| KY048224 | DSOPH2158 |
| FJ917336 | 196.1E.03 |
| KY048250 | DSOPH3159 |
| Genbank accession 16S | Sequence |
| KY048203 | Op1042_3E_1 |
|  | Op877_2E_9 |
| KY048209 | Op531_3E_12 |
|  | Op531_3E_13 |
|  | Op531_3E_4 |
|  | Op531_3E_6 |
|  | Op531_3E_8 |
|  | Op913_3E_10 |
| KY048214 | Op803_4C_1 |
|  | Op806_3C_2 |
| FJ917301 | 177.1E.01 |
|  | 177.1E.05 |
|  | 195.1E.07 |
| KY048204 | Op1042_3E_5 |
|  | Op877_2E_5 |
| FJ917305 | Op1042_3E_10 |
|  | Op1042_3E_6 |
|  | Op762_3C_2 |
|  | Op762_5C_1 |
|  | Op762_6C_1 |
|  | Op787_5C_3 |
|  | Op787_6C_3 |
|  | Op787_6C_4 |
|  | Op803_3C_2 |
|  | Op803_3C_3 |
|  | Op803_3C_4 |
|  | Op803_4C_2 |
|  | Op803_4C_3 |
|  | Op806_3C_1 |
|  | Op806_3C_3 |
|  | Op806_3C_4 |
|  | Op806_7C |
|  | Op806_8C_1 |
|  | Op806_8C_3 |
|  | Op806_8C_4 |
|  | Op818_2E |
|  | Op818_3C_1 |
|  | Op818_3C_3 |
|  | Op818_3C_5 |
|  | Op818_4C_1 |
|  | Op818_4C_2 |
|  | Op818_4C_3 |
|  | Op818_4C_4 |
|  | Op818_4C_5 |
|  | Op826_2E |
|  | Op826_3C_2 |
|  | Op843_2E |
|  | Op843_3C_1 |
|  | Op843_7C_1 |
|  | Op843_7C_2 |
|  | Op843_7C_4 |
|  | Op867_4E_1 |
|  | Op867_4E_3 |
|  | Op867_4E_5 |
|  | Op867_4E_7 |
|  | Op877_2E_7 |
|  | Op877_2E_8 |
|  | 312.3C.01 |
|  | 312.3C.03 |
|  | 312.3C.16 |
|  | 398.1E.12 |
|  | 422.1C.03 |
|  | 422.1C.06 |
|  | E82.2C.04 |
|  | E82.2C.05 |
|  | 312.3C.05 |
|  | 312.3C.15 |
|  | 398.1E.13 |
|  | 422.1C.02 |
|  | 422.1C.10 |
| FJ917306 | Op1042_3E_2 |
|  | Op1042_3E_3 |
|  | Op1042_3E_4 |
|  | Op1042_3E_9 |
|  | Op867_4E_10 |
|  | Op867_4E_2 |
|  | Op867_4E_6 |
|  | Op867_4E_8 |
|  | Op867_4E_9 |
|  | Op877_2E_1 |
|  | 312.3C.07 |
|  | 312.3C.09 |
|  | 422.1C.01 |
|  | 422.1C.04 |
|  | 422.1C.05 |
|  | 422.1C.08 |
|  | 422.1C.14 |
|  | E82.2C.01 |
|  | E82.2C.02 |
|  | 422.1C.07 |
| KY048206 | Op1042_3E_8 |
|  | Op877_2E_3 |
| FJ917291 | 57.3C.05 |
|  | 321.2C.04 |
| FJ917293 | Op913_3E_1 |
|  | Op913_3E_2 |
|  | Op913_3E_3 |
|  | Op913_3E_4 |
|  | Op913_3E_5 |
|  | Op913_3E_6 |
|  | Op913_3E_9 |
|  | Op914_3E_3 |
|  | Op914_3E_5 |
|  | Op914_3E_8 |
|  | Op914_3E_9 |
|  | Op917_3E_10 |
|  | Op917_3E_4 |
|  | Op917_3E_6 |
|  | Op917_3E_7 |
|  | 57.3C.10 |
|  | 57.3C.12 |
|  | 59.2C.03 |
|  | 59.2C.06 |
|  | 59.2C.07 |
| KY048208 | Op531_3E_11 |
|  | Op762_2E |
|  | Op787_5C_1 |
|  | Op787_5C_2 |
|  | Op787_5C_4 |
|  | Op787_6C_1 |
|  | Op787_6C_2 |
|  | Op787_6C_5 |
|  | Op803_3C_1 |
|  | Op803_3C_5 |
|  | Op803_3C_6 |
|  | Op806_2E |
|  | Op806_8C_2 |
|  | Op818_3C_2 |
|  | Op818_3C_4 |
|  | Op826_3C_1 |
| FJ917296 | Op877_2E_2 |
|  | Op895_3E_10 |
|  | 92.3C |
|  | 92.6C |
|  | 92.15C |
|  | 92.17C |
|  | 114.8C |
|  | 362.1C.09 |
|  | 398.1E.02 |
|  | 398.1E.14 |
|  | E43.2C.03 |
|  | 114.2C |
|  | 114.6C |
|  | 114.13C |
|  | 312.3C.02 |
|  | 398.1E.07 |
|  | E73.2C.06 |
|  | 114.11C |
| FJ917304 | 194.1E.03 |
|  | E73.2C.02 |
| FJ917294 | Op877_2E_10 |
|  | Op877_2E_6 |
|  | Op895_3E_3 |
|  | Op895_3E_4 |
|  | Op895_3E_8 |
|  | Op913_3E_7 |
|  | Op913_3E_8 |
|  | Op914_3E_2 |
|  | Op914_3E_6 |
|  | Op917_3E_1 |
|  | Op917_3E_3 |
|  | Op917_3E_5 |
|  | Op917_3E_8 |
|  | Op917_3E_9 |
|  | 57.3C.11 |
|  | 57.3C.13 |
|  | 92.10C |
|  | 196.1E.10 |
|  | 92.4C |
|  | 321.2C.01 |
|  | E73.2C.11 |
|  | 92.12C |
|  | 321.2C.03 |
|  | 321.2C.06 |
|  | 92.16C |
|  | 114.3C |
|  | 196.1E.07 |
|  | 177.1E.04 |
|  | 177.1E.10 |
|  | 177.1E.12 |
|  | 196.1E.01 |
|  | 196.1E.04 |
|  | 196.1E.06 |
|  | 196.1E.08 |
|  | 194.1E.01 |
|  | 194.1E.06 |
|  | 195.1E.06 |
|  | 195.1E.08 |
|  | 194.1E.02 |
|  | 195.1E.05 |
|  | 194.1E.04 |
|  | 194.1E.07 |
|  | 194.1E.08 |
|  | 194.1E.09 |
|  | 196.1E.03 |
|  | 321.2C.02 |
|  | 362.1C.07 |
|  | 398.1E.01 |
|  | 398.1E.15 |
|  | E73.2C.03 |
|  | E73.2C.12 |
|  | E82.2C.03  195.1E.03  195.1E.04 |
| FJ917302 | 177.1E.06 |
|  | 177.1E.09 |
|  | 177.1E.11  177.1E.02 |
| FJ917300 | Op877_2E_4 |
|  | Op914_3E_10 |
|  | 114.5C |
|  | E73.2C.10 |
| KY048213 | Op762_6C_2 |
|  | Op843_7C_3 |
| FJ917295 | Op895_3E_7 |
|  | 57.3C.14 |
|  | 92.9C |
|  | 92.14C |
|  | 362.1C.02 |
|  | 362.1C.03 |
|  | 362.1C.04 |
|  | 362.1C.05 |
|  | E73.2C.01 |
|  | 92.7C |
|  | 177.1E.07 |
|  | 196.1E.05 |
|  | 362.1C.01 |
|  | 362.1C.10 |
|  | 362.1C.11 |
|  | E73.2C.05 |
| FJ917299 | 92.11C |
|  | 92.13C |
|  | 114.4C |
|  | 114.7C |
|  | 114.10C |
|  | 362.1C.12 |
|  | E73.2C.09 |
| FJ917292 | Op917_3E_2 |
|  | 57.3C.08 |
| KY048210 | Op531_3E_5 |
| KY048211 | Op531_3E_9 |
| KY048207 | Op531_3E_10 |
| KY048212 | Op762_4C_1 |
| KY048205 | Op1042_3E_7 |
| FJ917298 | 92.8C |
| FJ917297 | 92.5C |
| FJ917303 | 194.1E.05 |
| KY048217 | Op914_3E_7 |
| KY048215 | Op895_3E_2 |
| FJ917308 | E73.2C.08 |
| FJ917290 | 57.3C.03 |
| KY048216 | Op895_3E_5 |

**Supplementary Figure 1.** Historical scenarios evaluated using Bayesian computation (ABC). In these scenarios t# represents time in generations and is based off the four genetic populations identified by STRUCTURE. **Scenario 1**. The three geographic regions split at approximately the same time with a more recent diversification in the Weddell Sea. **Scenario 2**. Initial separation of the Bellingshausen Sea with a subsequent split between the Ross with the most recent diversification in the Weddell Sea, consistent with a trans-Antarctic seaway hypothesis. **Scenario 3**. Diversification of all populations at approximately the same time, possibly due to isolation in refugium. **Scenario 4**. Initial separation of the Ross Sea with a much more recent diversification in the Bellingshausen and two in the Weddell Sea. **Scenario 5**. Initial separation of the Ross, then Bellingshausen and finally diversification in the Weddell, consistent with West to East distribution and diversification through the ACC. **Scenario 6**. An initial separation of Western and Easter Antarctica with later diversification in both regions. **Scenario 7**. Initial isolation of the Bellingshausen Sea and a much more recent diversification between the three remaining populations.


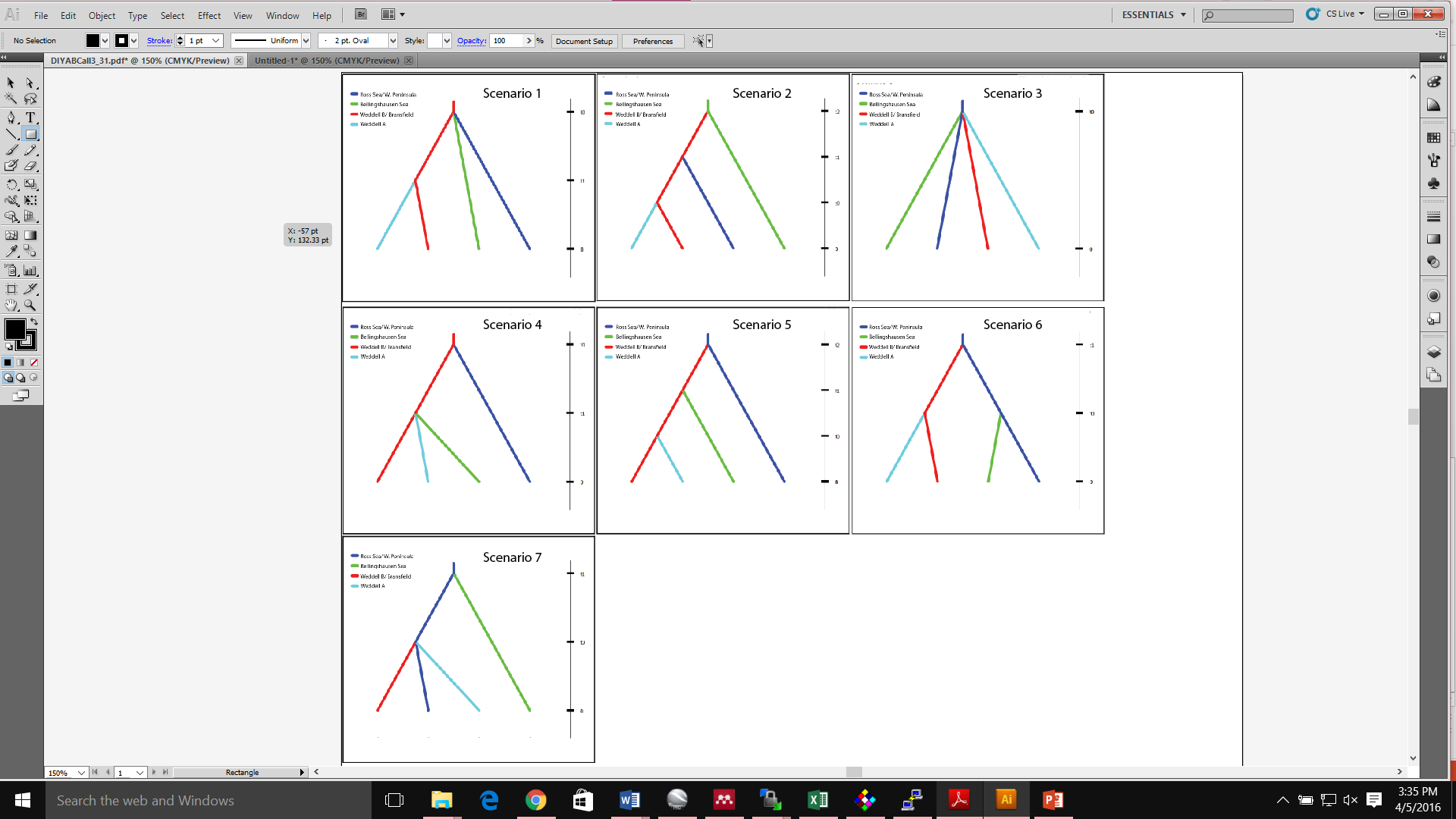


**Supplementary Figure 2.** STRUCTURE HARVESTER (Earl & vonHoldt 2012) average calculations of DeltaK based on the 1999 SNP loci.

**Supplementary Figure 3.** Patterns of population structure for *Ophionotus victoriae* based on SNP data analyzed in STRUCTURE 2.3.4. (Pritchard et al. 2000) and visualized in DISTRUCT (Rosenberg 2004) testing for the true number of populations (*K*). *K*=2 is presented in the graph above.
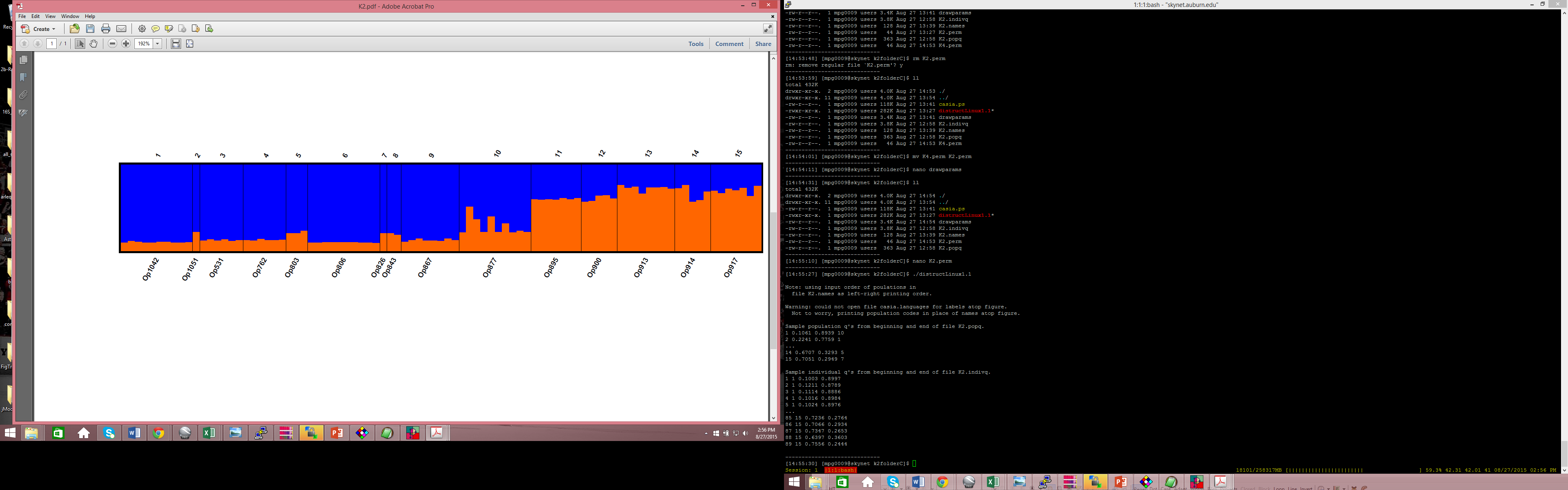


**Supplementary Figure 4.** Patterns of population structure for Ophionotus victoriae based on SNP data analyzed in STRUCTURE 2.3.4. (Pritchard et al. 2000) and visualized in DISTRUCT (Rosenberg 2004) testing for the true number of populations (K). K=8 is presented in the graph above.


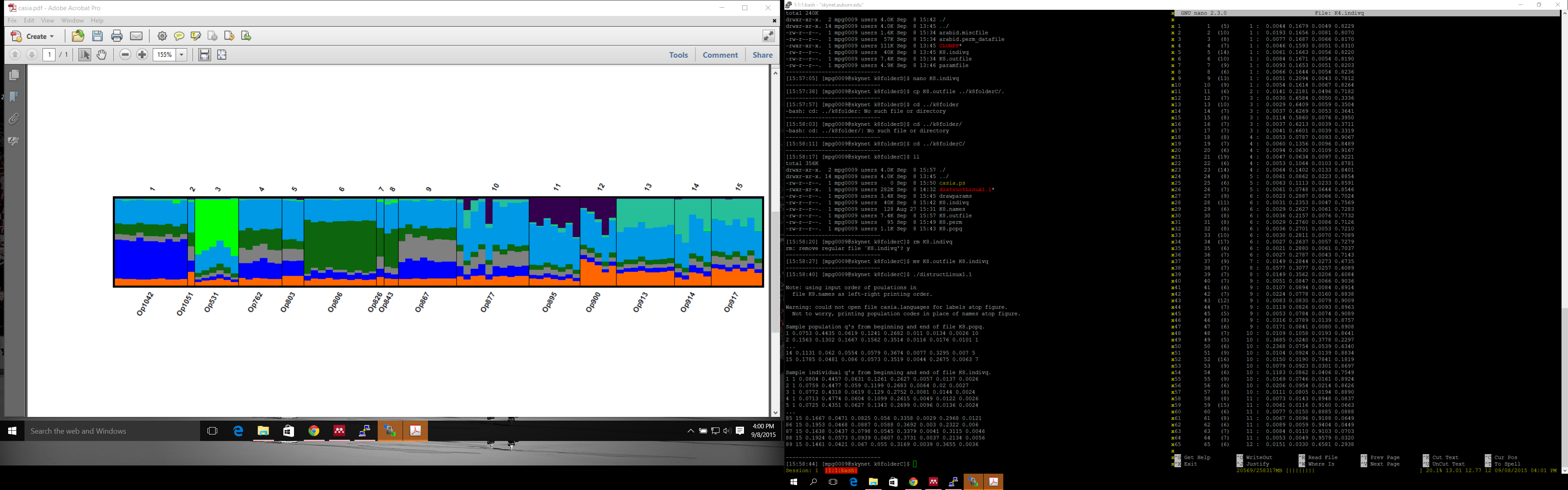


**Supplementary Figure 5.** PCA results based on SNP data for samples labeled by the geographic regions of the Ross Sea, Bellingshausen Sea, western Antarctic Peninsula, Bransfield Straits and Weddell Sea. Sampling locality Op867 was at the boundary of the western Antarctic Peninsula and Bransfield Strait and is likely bathed in Weddell Sea water coming through Antarctic Sound and southwest along continental edge of the Peninsula. Thus for Op 867 samples, we have circled the gray X used to denote western Antarctic Peninsula samples as they appear to cluster themselves with either other samples further southwest on the Antarctic Peninsula or with samples from the Weddell Sea.


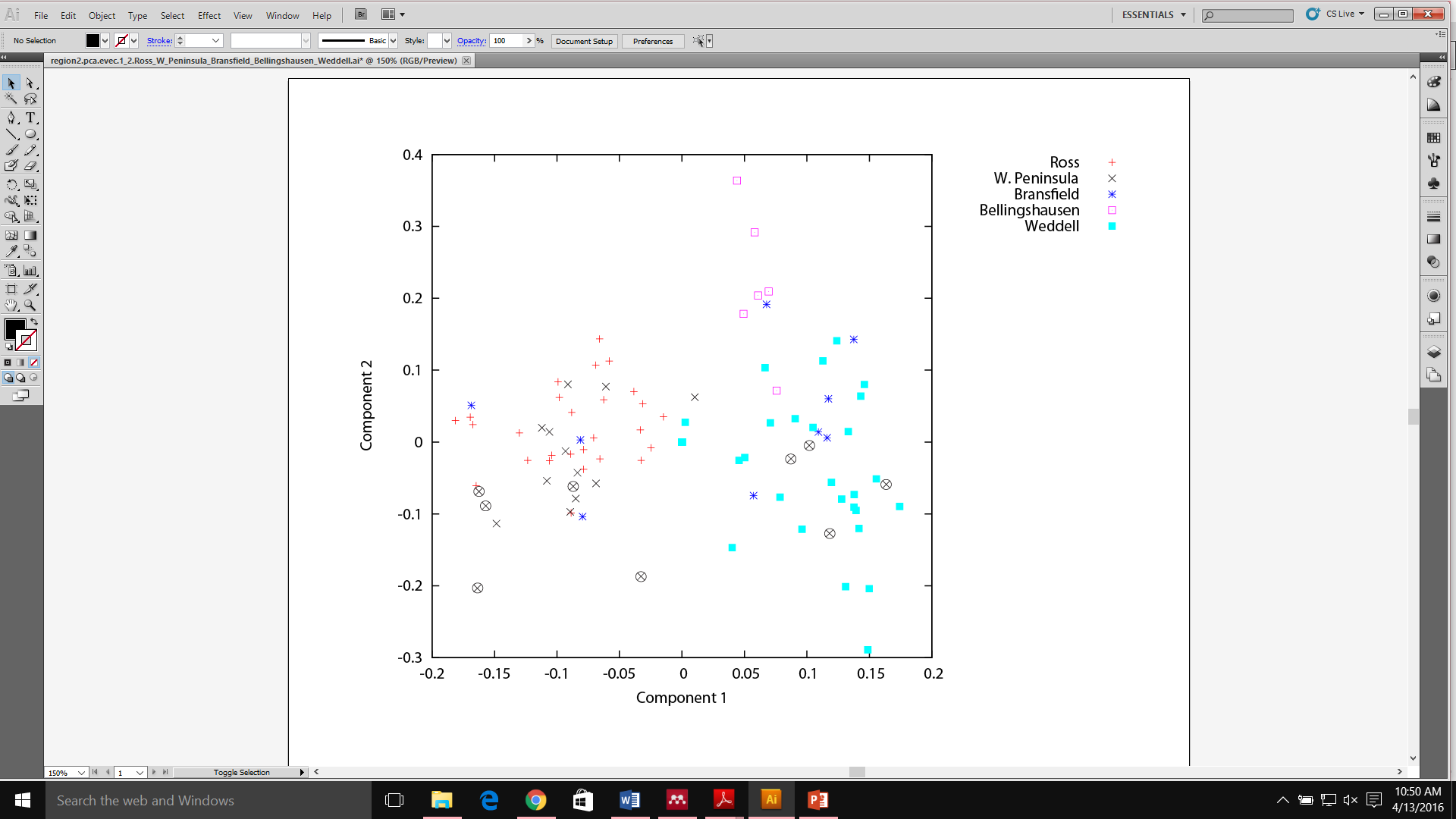


**Supplementary Figure 6.** Maximum likelihood analysis of COI mitochondrial sequences for 414 individuals of *O. victoriae*.
