# Supplementary figures and images for: Geographic structure in the Southern Ocean circumpolar brittle star Ophionotus victoriae (Ophiuridae) revealed from mtDNA and single‐nucleotide polymorphism data
Source: Ecol Evol. 2016 Dec 16;7(2):475–85. doi: 10.1002/ece3.2617 (PMC5243193; doi:10.1002/ece3.2617)

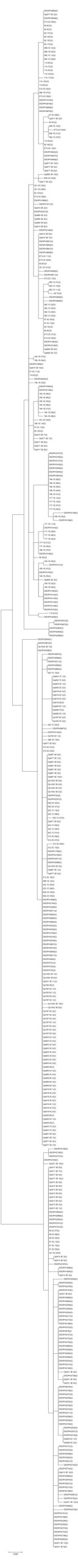

Supplement: Supplementary file 2 [file ECE3-7-475-s002.PDF]
